# Devising a Zfu2p targeted antifungal strategy that may preserve commensalism while suppressing *Candida albicans* virulence during vulvovaginal candidiasis

**DOI:** 10.1038/s41598-026-46848-5

**Published:** 2026-05-13

**Authors:** Ali Rejwan Kabir, Anis Ahmad Chaudhary, Joydeep Chakraborty, Soumita Podder

**Affiliations:** 1 Computational Systems Biology Laboratory, Department of Microbiology, Raiganj University, Uttar Dinajpur, Raiganj, West Bengal 733134 India; 2 Department of Biology, College of Science, Imam Mohammad Ibn Saud Islamic University (IMSIU), 11623 Riyadh, Saudi Arabia; 3 Cell Biology and Bacteriology Laboratory, Department of Microbiology, Raiganj University, Uttar Dinajpur, Raiganj, West Bengal 733134 India

**Keywords:** *Candida albicans*, Vulvovaginal candidiasis, Zfu2p, Ergotamine, Master regulator, Drug repurposing, Computational biology and bioinformatics, Diseases, Microbiology

## Abstract

*Candida albicans* is the leading cause of vulvovaginal candidiasis (VVC), a multifactorial disease of the lower female reproductive tract. Despite numerous studies, the molecular-pathophysiology during VVC and its commensal state within the host remains elusive. To address this, we performed a comprehensive computational analysis by integrating RNA-Seq datasets from both host and pathogen. Differentially expressed genes were identified and used to construct three network models: within the pathogen, host, and host-pathogen interaction. Subsequent inter-network nodes mapping enabled us to attain two distinct condition-specific fungal targeted networks in the host, during VVC (466 nodes and 496 edges) and commensal state (162 nodes and 176 edges), which were validated by condition-specific immune response by gene ontology analysis. Intending to target pathogenesis while preserving its commensal integrity, we identified an exclusive fungal protein Zfu2p that acts as a master regulator in the gene regulatory network of the pathogen. It promotes pathogenic attributes through its target gene *RNR22* and interacts with the host protein UBC. Subsequently, the drug repurposing strategy coupled with molecular dynamics analysis identified Ergotamine as a promising candidate, which may disrupt biofilm formation while preserving commensal balance. This study offers insights into VVC pathophysiology and suggests a targeted therapy, though experimental validation of Ergotamine’s efficacy remains essential.

**Supplementary Information:**

The online version contains supplementary material available at 10.1038/s41598-026-46848-5.

## Introduction

Vulvovaginal candidiasis (VVC) is the most common fungal infection in women and is mainly caused by *Candida albicans (C. albicans).* It is an opportunistic pathogen and carries a perplexing ability to adapt to different host niches. *C. albicans* survives as a commensal in healthy individuals enriched with yeast form^1^. Moreover, *C. albicans* shows its phenotypic switch ability and converts from yeast to hypha form, which could be considered as a pathogenic attribute^1^ depending on the host environment^2^, i.e., CO_2_ concentration, iron and phosphate limitation, pH, temperature, oxidative stress, nitrosative stress, etc ^3,4^. *Candida species*, however, can cause infections or turn asymptomatic colonizers. They are found in distinct niches of the human body and cause superficial to systemic infection. Moreover, recurrent vulvovaginal candidiasis (RVVC) affects around 138 million annually, and it has been predicted that it will reach the mark of 158 million cases per year^5^. Additionally, in developing countries like India, the prevalence of RVVC is about 24.3 million^6^, which depicts a perilously alarming situation, and suggests that the present treatment is not significant enough to ameliorate the situation. VVC refers to symptomatic inflammation in the vagina region and is primarily caused by a fungal infection, primarily due to *C. albicans*^7^. Additionally, secreted aspartyl proteins (SAPs) and the HWP1 gene of the *C. albicans* were upregulated during VVC and degrade E-Cadherin, complement pathway components, and histatin in the host, allowing them to overcome host defence^8,9^. Moreover, in response to VVC infection, neutrophils and NLRP3 inflammasome are recruited by the host^10–12^. Additionally, neutrophils and Th17 were the prime host response in the clearance of *C. albicans* in the vaginal region^13,14^. Consequently, an earlier study had discerned that recruited neutrophils appear to be ineffective at diminishing fungal load and invasion; as a result, they seem to aggravate the symptoms associated with vaginitis rather than providing defence against the disease^7^. Therefore, the molecular mechanism behind the pathogenesis of vulvovaginal candidiasis, host response, and its aetiology are still under wraps or delusional as it has perpetually evolved with us. Most of the research has been carried out to investigate the pathogenic repertoire of *C. albicans* during VVC and immunological response offered by the host^11,12,15–17^. Current antifungal drugs for VVC primarily target pathogenicity, overlooking the beneficial commensal role of *C. albicans* within the host. It has been reported earlier that it helps both systemic and mucosal-associated responses. Literature evidence divulged that it helps in calibrating Th17 response, which protects from *Clostridium difficile* infection by upregulating IL17A during *C. albicans* colonization^18^, while colonization of *C. albicans* in neutropenic mice helps to impede *Pseudomonas aeruginosa* infection by suppressing siderophores biosynthesis, i.e., Pyochelin and Pyoverdine^19,20^. Interestingly, this protection extends beyond disseminated candidiasis, as demonstrated by induced cross-protection against unrelated pathogens like *Staphylococcus aureus*, and other systemic pathogens in the gut^21–23^. Additionally, it also helps to train host immunity^24,25^, which has been clearly accentuated in an earlier report that mice lacking adaptive immunity due to a deficiency in T and B lymphocytes, but an initial exposure to *C. albicans* prevents death and also offers defence against subsequent *C. albicans* infections^26^.

Therefore, we require a robust protocol to understand the tripartite or banal existence of *C. albicans* in the microbiome and design an efficient drug to curb the infection. Presently, drug options available might not be effective enough to eradicate the infection because one of the main reasons is that during the identification of a target, we ignore the banal associates’ effects of *C. albicans*, which aid the host in numerous ways. Thus, antibiotic or antifungal drugs we use to treat fungal infection cause dysbiosis in the distinct niches^27,28^. Moreover, the availability of antifungal options to treat *C. albicans* infections nowadays is already restricted due to the limited types of drugs available, including azoles, polyenes, echinocandins, and pyrimidine analogs. Subsequently, numerous in silico approaches, i.e., protein-protein interaction, drug repurposing, structure‐based docking, pharmacophore modelling, and quantitative structure activity relationship (QSAR) analysis for drug discovery, have been developed to address this issue^29–31^. Of these, protein-protein interaction network and drug repurposing approach is a new branch of computational science that utilizes approved drugs for the treatment of different diseases^30,31^. Instances encompass food and drug administration (FDA) approved drugs “5 fluorouracil” used as an anticancer, “tamoxifen” for breast cancer, “saquinavir” for HIV/AIDS, and “hydroxychloroquine” for malaria, “disulfiram” utilized for alcohol dependence, all of which have been repurposed effectively as potent treatments for infections caused by viruses, bacteria, fungi, and parasites^32–37^. This highlights that drug repurposing holds significant promise as an emerging approach for rapidly identifying safe, approved, and readily available compounds suitable for the effective treatment of infectious diseases, including candidiasis^38^.

In this present communication, we were intrigued to determine the proteins that were exclusively present in pathogenic conditions and play a key role in pathogenesis, with minimal effect on commensal aid provided to the host. Comparing the commensal and pathogenic sub-network of *C. albicans* in the host, we have revealed an exclusive master regulator Zfu2p controlling transcriptional regulation of the pathogenic network. Moreover, we have employed the drug repurposing strategy to devise promising antifungal drugs from FDA approved drugs. The proposed drug Ergotamine could potentially provide an alternative means for controlling fungal infections caused by other species as well, which are the leading cause of VVC.

## Result

### Reconstruction of the integrated gene regulatory network of *C. albicans* during pathogenic conditions

*C. albicans*, having the potential to colonize the mucosal surfaces of the vaginal region or inanimate substances like a catheter, leads to nosocomial infections and causes VVC. To delve into the molecular pathogenic repertoire of *C. albicans* during VVC, we extracted DEGs of *C. albicans* from a supplementary of RNASeq data analysis by Manczinger et al., *GSE54694* ^39^. We made a list of 1541 pathogenic differentially expressed genes (DEGs) (Supplementary Table 1) and reconstructed a pathogenic protein-protein interaction network (pPPIN), consisting of 598 nodes and 2650 edges, applying the *STRING database v11.5* (Supplementary Fig. 1A)^40^. Afterwards, we performed biological, cellular, and molecular function enrichment analysis by utilizing *ClueGO*^41^, a Cytoscape plugin v3.10.0, to validate their functional roles in the pPPIN network. We observed the enrichment of several functions essential for pathogenicity in the host, i.e., “filamentous growth of a population of unicellular organisms” (GO:0044182), “cellular response to nutrient levels” (GO:0031669), “P-type ion transporter activity” (GO:0015662), “cellular response to starvation” (GO:0009267), and so forth (Supplementary Fig. 2A**)**.

**Fig. 1:**
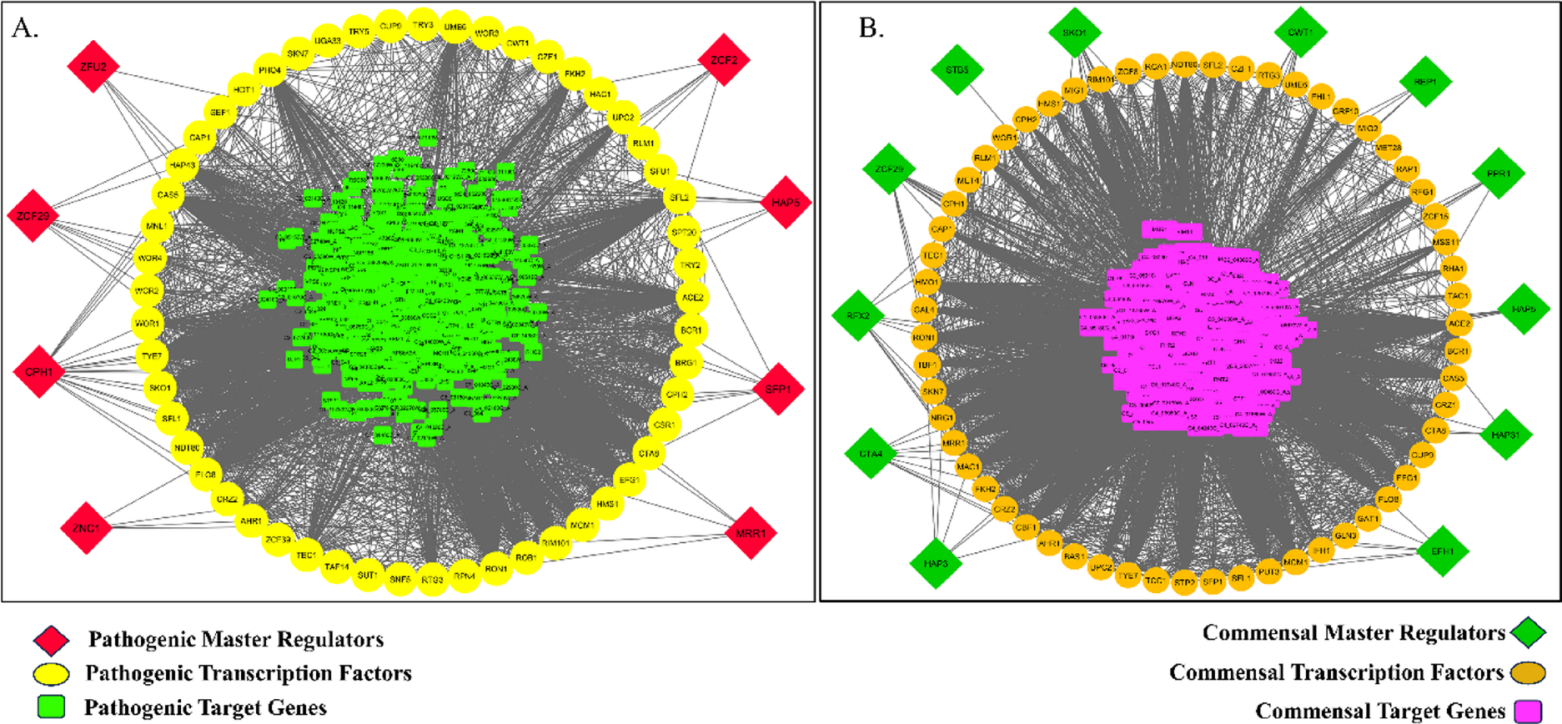
Gene regulatory networks of *C. albicans* in the vaginal niche during VVC and commensal conditions: (**A**) The pathogen gene regulatory network (pGRN) consists of 598 nodes and 4285 edges, including 61 transcription factors (TFs) and 537 target genes (TGs). (**B**) The commensal gene regulatory network (cGRN) includes 793 nodes and 7331 edges, with 71 transcription factors and 722 target genes.

Since the transcriptional circuit governs gene expression and regulation in response to distinct environmental cues, to determine the transcription factors (TFs) responsible for regulating the 598 nodes, which considered as target genes (TG) within the pPPIN, we constructed the gene regulatory network (GRN), which consists of three types of interactions: TF-TG, TF-TF, and TG-TG. We looked for the TFs that regulate gene expression during biofilm formation and various stress circumstances by using the *Pathoyeastract database* because it has been demonstrated that biofilm development and different stress conditions are critical for vaginal candidiasis^2^. Finally, we built a pathogenic gene regulatory network (pGRN) in which 61 pathogenic transcription factors (pTFs) control the expression of 537 pathogenic target genes (pTGs) (Fig. 1A). Moreover, GO biological, cellular, and molecular functional enrichment analysis of pGRN showed that “biofilm formation” (GO:0042710), “cellular response to metal ion” (GO:0071248), “cellular response to stress” (GO:0033554), “copper ion binding” (GO:0005507), “methionine metabolic process” (GO:0006555), “regulation of phenotypic switching” (GO:1900239) etc. (Supplementary Fig. 2B**)**. These functional enrichment analyses clearly depicted that the proteins involved in the pPPIN and pGRN of *C. albicans* play a key role in biofilm formation, which helps to establish pathogenic repertoire in the vaginal region, leading to VVC^42^. Additionally, methionine metabolism was observed to play a vital role in the virulence of *C. albicans*^43^, phenotypic plasticity, and stress-induced pathogenesis in earlier studies^3^, and has also been implicated in the development of vulvovaginal candidiasis.

Next, we were interested in identifying the master regulator (MR) in the pGRN transcriptional orchestra. To create pyramid-shaped hierarchical structures, we have constructed the network with three different types of interactions- TF-TF, TF-TG, and TG-TG, and determined the hierarchy index (v) of the TFs involved (Fig. 1A). According to their rank of v, the structures were designed so that the majority of TFs remain at the lower level, and just a few master TFs are at the top. We have identified 8 pathogenic master regulators (pMR), i.e., Znc1p, Cph1p, Hap5p, Mrr1p, Sfp1p, Zcf2p, Zcf29p, and Zfu2p, in the regulatory network of the pGRN.

### Reconstruction of the integrated gene regulatory network of *C. albicans* during commensalism

The commensalistic lifestyle and molecular mechanism of *C. albicans* in the vaginal region are yet to be visualized and elucidated clearly. Therefore, to decipher the commensalistic molecular repertoire of *C. albicans* in the vaginal region, we extracted DEGs of *C. albicans* from a supplementary of a RNASeq data analysis by Manczinger et al., *GSE54694*^39^. We retrieved a list of 1547 commensal DEGs, which help to study the adhesion and colonization of *C. albicans* **(**Supplementary Table 1**)**, and consequently, constructed a commensalism specific protein-protein interaction network (cPPIN) consisting of 824 nodes, which considered as commensal target genes (cTGs) and 5856 edges, using the *STRING database v11.5* (Supplementary Fig. 1B)^40^. Furthermore, we performed GO biological, cellular and molecular function analysis of cPPIN to establish the role of proteins involved in this network and we discerned that “regulation of growth” (GO:0040008), “DNA-binding transcription factor activity” (GO:0003700), “yeast-form cell wall” (GO:0030445), “development of symbiont in host” (GO:0044114), “carbohydrate homeostasis” (GO:0033500), “glucose homeostasis” (GO:0042593), “DNA strand elongation” (GO:0022616) and so forth, functions were enriched (Supplementary Fig. 2C**)**.

Afterward, we also constructed the commensalism specific regulatory network (cGRN), following a similar way described in the previous section. We looked for the commensal transcription factors (cTFs) that regulate gene expression during commensal circumstances (i.e., log phase growth) by using the *Pathoyeastract database*. Finally, we reconstructed a commensal gene regulatory network (cGRN), with 793 nodes and 7331 edges in which 71 TFs control the expression of 722 TGs (Fig. 1B). Furthermore, GO biological, cellular and molecular function enrichment of cGRN showed the preponderance of “(1->3)-beta-D-glucan biosynthetic process” (GO:0006075), “replication fork protection complex” (GO:0031298), “DNA binding” (GO:0003677), “cell cycle process” (GO:0022402), “quorum sensing” (GO:0009372), “development of symbiont in host” (GO:0044114), “intracellular iron ion homeostasis” (GO:0006879), “chlamydospore formation” (GO:0001410) etc. (Supplementary Fig. 2D**)**. These function enrichment analyses strongly divulged that the proteins involved in the cPPIN and cGRN of *C. albicans* play a key role in normal growth, homeostasis between the macro and micro molecules. Additionally, quorum sensing helps to inhibit the hyphal formation of *C. albicans*^44^, and it is well acknowledged that hyphal formation is a pathogenic attribute during VVC infection^45,46^.

Next, we were intrigued to identify MRs in the commensal gene regulatory network (cGRN) in a similar way to that applied for pGRN (Fig. 1B). We have identified 12 commensal master regulators (cMR), i.e., Hap3p, Cta4p, Cwt1p, Efh1p, Hap5p, Ppr1p, Rep1p, Rfx2p, Sko1p, Stb5p, Zcf29p, and Hap31p in the regulatory network of the cGRN.

### Identification of potential *C. albicans* targets in the human proteome

**Fig. 2:**
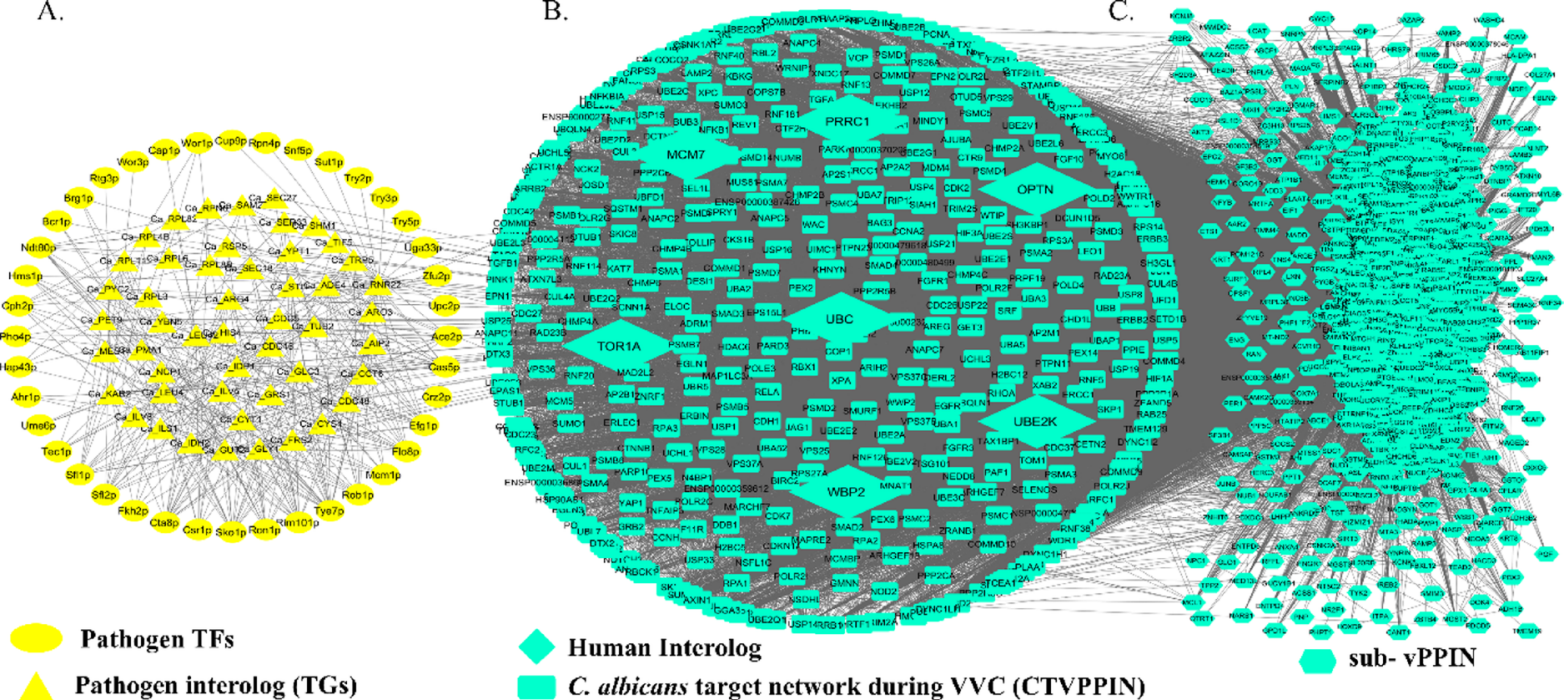
Target network (CTVPPIN) of *C. albicans*during VVC in the host. (**A**) Yellow circular nodes depict pathogen transcription factors (pTFs) and a yellow triangle represents pathogenic target genes (pTGs) of the pathogen, present in pathogen interolog nodes. (**B**) Teal color nodes represent host nodes. The central network is the VVC state target network, i.e., CTVPPIN, which consists of 466 nodes and 496 edges. 7 rhombus shaped teal color host nodes interolog nodes, that are direct interactors of pathogen proteins, facilitating host and pathogen interaction during the VVC niche. (**C**) Hexagon represented by sub-vPPIN nodes.

**Fig. 3:**
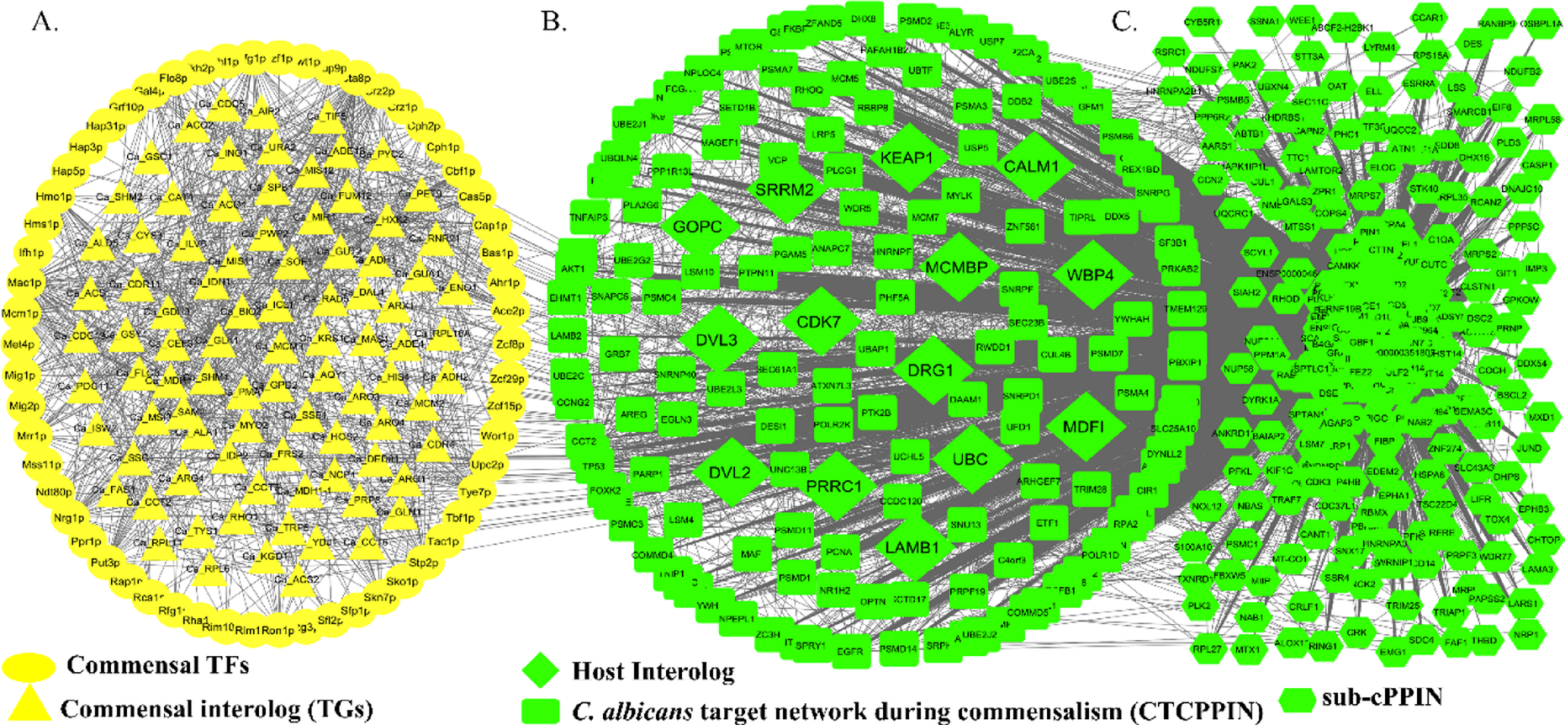
Target network (CTCPPIN) of *C. albicans* during commensal condition in the host. (**A**) Yellow circular nodes depict commensal transcription factors (cTFs) and yellow triangle-shaped nodes represent commensal target genes (cTGs) of the pathogen, present in the pathogen interolog node. (**B**) Green color nodes represent host nodes. In the middle, a commensal state target network, i.e., CTCPPIN, comprising 162 nodes and 176 edges. 14 green color rhombus nodes are host interolog nodes, directly interacting with commensal pathogen proteins, facilitating host and pathogen interaction during the commensal condition niche. (**C**) Hexagon represents sub-cPPIN nodes.

We have retrieved 317 *C. albicans* homologs that interacted with 119 human proteins, consisting of 432 PPIs by applying interolog approach, from our previous study^47^. In the following stage of the in-silico workflow, we attempted to isolate those *C. albicans* proteins from the interologs that might be involved in mediating the vulvovaginal candidiasis and establishing commensal conditions reported in the previous section. To pursue, we mapped 317 *C. albicans* interologs with 598 pGRN and 793 cGRN proteins of *C. albicans*, which have been linked to VVC and commensal circumstances, respectively. We have identified a set of 49 proteins that are involved in pathogenic conditions and 89 proteins that are associated with commensal conditions. These proteins belong to distinct pools and play significant roles in their respective contexts. Thus, it could be stated that these 49 proteins might help *C. albicans* to establish a pathogenic repertoire in the vagina region of the host, which leads to VVC. Similarly, 89 proteins help in establishing a commensal state in the vaginal region. To acquire corresponding human proteins, we mapped these *C. albicans* proteins to their interologs. In the meantime, we observed that 13 human proteins directly interact with 49 pathogenic *C. albicans* proteins, and 27 human proteins that interact with 89 commensal *C. albicans* proteins. These interactions contribute to a total of 58 and 100 protein-protein interactions (PPIs) in the vaginal region during VVC and commensal conditions, respectively (Figs. 2 and 3). Consequently, it is highly intriguing to explore and investigate the molecular interactions between the 13 human proteins and the 49 pathogenic *C. albicans* proteins, as well as the interactions between the 27 human proteins and the 89 commensal *C. albicans* proteins.

### Reconstruction of the *C. albicans* targeted network in the host in a pathogenic condition

It is generally acknowledged that vaginal candidiasis is caused by *C. albicans* infection in the vaginal area. With respect to other species, the risk is considerably high and is marked by surface tissue penetration, mediated by hyphae and fungal overgrowth. To delve into the pathophysiology triggered by the *C. albicans* targeted 13 human proteins, it is necessary to gather information about their interaction with vagina specific PPIN. Thus, we reconstructed the vagina-specific protein-protein interaction network (vPPIN) in the host comprising 5879 nodes and 80,386 edges following the protocol described in the Materials and Methods Section (2.5). Next, to validate and screen the host protein expression in vPPIN nodes during VVC, we utilized an RNASeq dataset *GSE207081*, in which a monolayer of A-431 vaginal epithelial cells was challenged with a VVC strain (CA01887)^48^. Subsequently, we mapped 5879 nodes of vPPIN with 2213 DEGs procured from the RNAseq dataset (Supplementary Table 2). We obtained 4848 nodes and 25,729 edges from vPPIN (Fig. 2C), in which either of the interacting nodes was a DEGs, and we considered it as a sub-vPPIN. Afterwards, we mapped 4848 nodes with 13 proteins of the host that directly interact with 49 *C. albicans* proteins (Fig. 2A) during pathogenesis. Finally, we acquired 7 out of 13 interolog host proteins that directly interact with a *C. albicans* targeted VVC specific PPIN (CTVPPIN), in which either of the connecting nodes interacts with *C. albicans* target proteins CTVPPIN, comprising 466 nodes and 498 edges, is vagina specific; thus, it could be inferred that this protein interactome might be implicated during vaginal candidiasis (Fig. 2B).

**Fig. 4:**
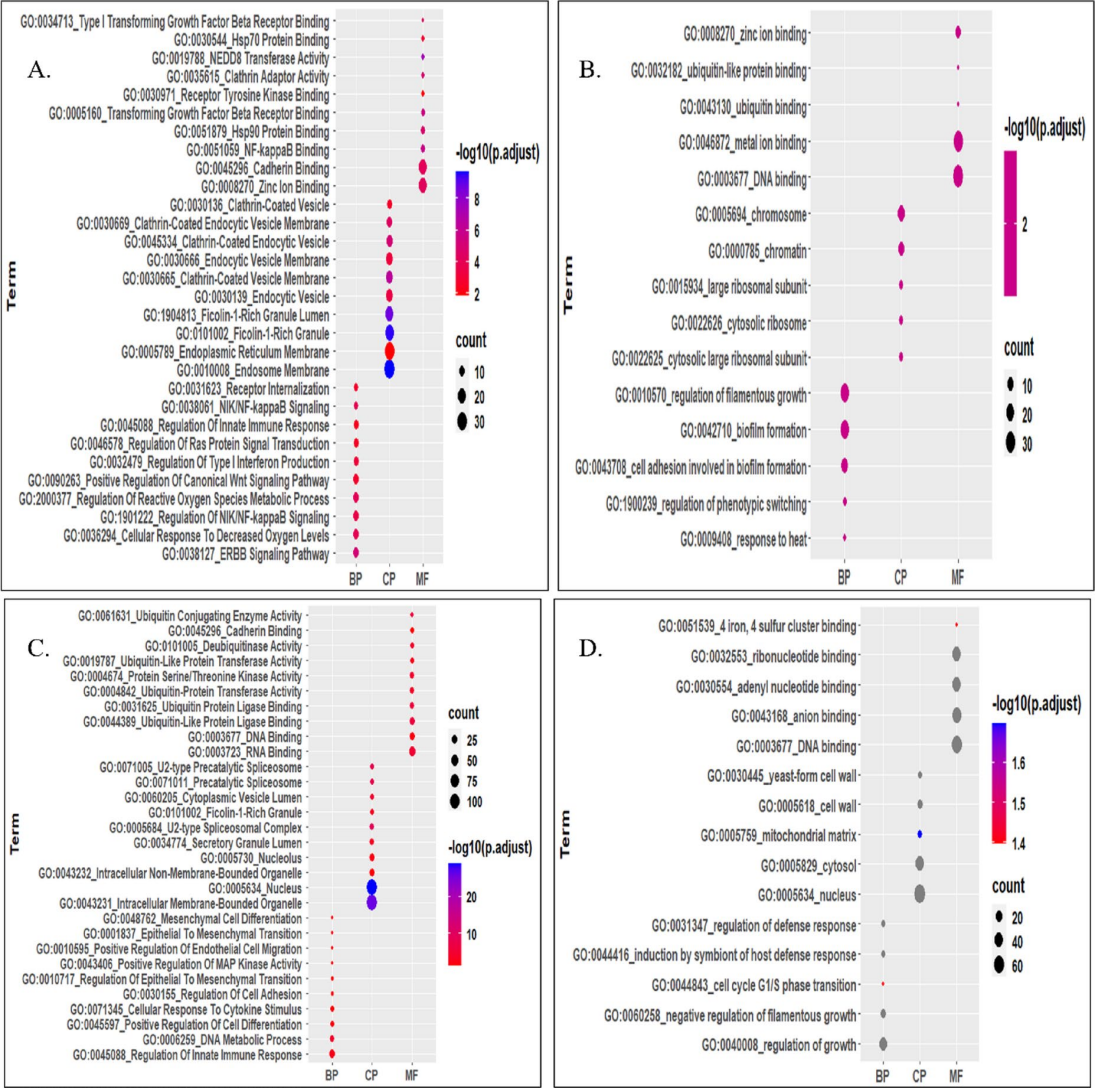
Dot plot of gene ontology analysis of target networks CTVPPIN and CTCPPIN. (**A**) For CTVPPIN (Host Axis). (**B**) For CTVPPIN (Pathogen Axis). (**C**) For CTCPPIN (Host axis). (**D**) For CTCPPIN (Commensal Axis). The count is represented by the size of the circle, which represents the number of genes that encode that particular function, and the color shade represents the level of significance of the function term. Here, BP- Biological Process, CP- Cellular Process, and MF- Molecular Function.

We performed GO biological, cellular, and molecular function enrichment analysis of host as well as fungal proteins participating in CTVPPIN by using *Enrichr*^49^ and ClueGO^41^, respectively (Fig. 4A), and clearly demonstrating that the CTVPPIN is enriched with several crucial immunological functions like “cellular response and signaling mediated by interleukin 6”, “NF-kappaB Signaling”, “Cytokine-Mediated Signaling Pathway”, which confer the status of host proteins during the pathogen infection^50^. Subsequently, we observed that pathogenic proteins are involved in primary biofilm maintenance and regulation of different stress conditions **(**Fig. 4B**).**

### Reconstruction of the *C. albicans* potential commensal condition targeted network in the host

It is generally acknowledged that the commensal lifestyle of *C. albicans* in the vaginal area is marked by adhesion and colonization, with an abundance of yeast-shaped cells, so that the host immune response can distinguish between yeast and hyphal morphology. To explicate the commensal association (primarily *C. albicans* adhesion and colonization, which not harming host) between host and *C. albicans*, we followed a similar methodology to construct a target network in pathogenic conditions. After constructing a vagina-specific network, we attempted to extract those nodes from vagina specific PPIN that were expressed in vaginal cells during adhesion and colonization of *C. albicans* in the commensal state. For that, we extracted 2144 DEGs from an RNAseq dataset *GSE207081*, in which a monolayer of A-431 vaginal epithelial cells challenged with a colonizing strain (CA14314)^48^**(**Supplementary Table 2**)**. Then, we mapped those DEGs with either of the nodes of vagina specific PPIN, consisting of 5879 nodes, and procured 4550 nodes and 23,093 edges from vagina specific PPIN, and we considered it as a commensal specific vPPIN nodes because they were expressed during the colonizing stain infection (Fig. 3C). Next, to identify the target network of *C. albicans* proteins that help in adhesion and colonization, we mapped 4550 nodes with 27 proteins of the host that directly interact with 89 *C. albicans* proteins (Fig. 3A) in the commensal state mentioned in the previous Section (2.4), which might help in adhesion and colonization. We acquired 14 out of 27 interolog host proteins that directly interact with a *C. albicans* targeted commensal PPIN (CTCPPIN), comprising 162 nodes and 176 edges (Fig. 3B).

Afterwards, GO biological, cellular, and molecular function enrichment analysis of host proteins by using *Enrichr*^49^**(**Fig. 4C**)** clearly depicted that the CTCPPIN is enriched with several crucial immunological functions like “Activation of Protein Kinase Activity” (GO:0032147), “Regulation of Innate Immune Response” (GO:0045088). Moreover, “positive regulation of MAP Kinase activity” (GO:0043406), which carries the ability to identify the yeast form with the help of c-Jun^51^. This confers the potentiality of the host to distinguish phenotypic plasticity and maintain homeostasis with the opportunistic pathogen. From the aforementioned result, it could be confirmed that the protein helps in establishing commensal repertoire in the vagina, and the gene ontology analysis also clearly indicates the immunological response from the host, which helps in tolerating the *C. albicans* commensal form. Furthermore, the functional enrichment analysis of *C. albicans* proteins involved in CTCPPIN showed enrichment for primary yeast cell formation and negative regulation of the filamentation process (Fig. 4D).

### Explicating the molecular link between the host and opportunistic pathogen during both conditions

Now, deciphering the molecular crosstalk between *C. albicans* pathogenic and commensal genes with human genes would provide a holistic view to know the exact scenario that transpires during VVC and commensalistic lifestyle in the vaginal region. We received a *C. albicans* target network during pathogenic condition i.e., CTVPPIN, which consists of 466 nodes and 496 edges, and exhibits some crucial immunological functions to impede the fungal infection as observed by GO functional analysis. Meanwhile, out of 466 nodes, 7 nodes (MCM7, OPTN, PRRC1, TOR1A, UBC, UBE2K, WBP2) of this network were found to be directly interacting with 48 *C. albicans* genes or pTGs. Interestingly, analysing the pGRN of the pathogen, we found that 48 pTGs were controlled by 40 pTFs. Furthermore, we observed that 48 pTGs were significantly upregulated in the DEG extracted from supplementary of RNAseq data analysis by Manczinger et al., *GSE54694*, and among 40 pTFs, 8 and 2 were upregulated and downregulated, respectively. In the midst of these 8 upregulated pTFs, Zfu2p was found to be upregulated and exhibited in the pMRs of the transcriptional cascade implicated for pathogenesis.

Similarly, we retrieved a *C. albicans* target network during commensal condition i.e., CTCPPIN, which consists of 162 nodes and 172 edges. Furthermore, gene ontology analysis has shown several pivotal cellular functions that help to set up a commensal repertoire in the vaginal region **(**Fig. 3**).** Meanwhile, we observed that among 162 nodes, 14 nodes (CALM1, CDK7, DRG1, DVL2, DVL3, GOPC, KEAP1, LAMB1, MCMBP, MDFI, PRRC1, SRRM2, UBC, and WBP4) were directly interacting with 81 genes of *C. albicans*. Among these 81 genes of *C. albicans* or cTGs, 69 genes were upregulated and 11 were downregulated. Investigating cGRN, we revealed that expression of these 81 cTGs was controlled by 58 cTFs, among which 22 and 1 cTFs were up- and downregulated, respectively, in the DEGs extracted from the supplementary of RNAseq data analysis by Manczinger et al., GSE54694. Interestingly, among these 22 upregulated cTFs, 7 (Cwt1p, Hap31p, Hap3p, Hap5p, Ppr1p, Sko1p, and Zcf29p) were included in the cMR list, which indicates their key role in governing the downstream gene during commensal lifestyle in the vaginal niche.

### Identification of Zfu2p protein as a potential target that retrogrades the pathogenic property of *C. albicans*

Being an opportunistic pathogen, it becomes challenging to find a potential target for *C. albicans*. We thus planned to select such a target that is exclusive for its pathogenicity but not at all involved in commensalistic association in the vagina. For this, we compared between pGRN and cGRN. Since the master regulator holds the highest position in a transcriptional orchestra, we were interested to find out such an exclusive master regulator that is present in pGRN and also does not act as a TF in cGRN. In this way, we have identified 3 unique pMR (Zfu2p, Znc1p, and Zcf2p). Afterward, we checked their expression in the pathogenic repertoire, and divulged that only Zfu2p upregulated significantly. Therefore, targeting Zfu2p as an antifungal drug target might help to diminish the pathogenic property of *C. albicans* during VVC without affecting its commensal properties. Subsequently, searching for a transcription factor as a drug target could be more beneficial since a single target could manipulate the expression of its downstream genes.

From our further investigation and literature survey, we found that the functional domain of the Zfu2p protein has a particular region of a 9–44 amino acid stretch^52,53^. Next, we were also interested in delving into the homologous proteins of Zfu2p, if present in other fungal pathogenic species, which can cause VVC. From the *Candida genome database* (CGD), we found that Zfu2p has homologs in 6 non-albicans and 1 aspergillus species [*Candida dubliniensis* CD36 (Cd36_87120), *Candida parapsilosis* CDC317 (CPAR2_602060), *Candida famata* (DEHA2D18810), *Candida guilliermondii* (PGUG_05199), *Candida glabrata* CBS138 (CAGL0K11902), *Candida auris* (B9J08_000794), and *Aspergillus nidulans* (AN5924)]. Multiple sequence alignment of the functional domain of Zfu2p with these homologous species showed considerable conservation among them (Supplementary Fig. 3), which increases the efficacy of prioritizing Zfu2p as a potential drug target. Additionally, it is worth mentioning that no homologs of Zfu2p were found to be present in humans as the domain contain Zn2Cys6 domain which specific to fungi^52,54^.

### Structure prediction and validation of Zfu2p protein

In the absence of an experimentally determined 3D structure, the priority for limiting the proper functioning of the aforementioned proteins is in-silico modelling of targets. Thus, we generated the 3D model by applying AlphaFold3^55^. Nowadays, AlphaFold structures have been well established, which revolutionized protein structure prediction and can be used as a template for 3D model building^56^. The generated model was also employed in GalaxyWeb^57^ for structural refinement, and we prioritized the best refined model for further analysis. Next, we applied several online tools to validate our 3D generated model. *PROCHECK* (Supplementary Fig. 4A**)** and *SWISS Model Server* were used to examine the Ramachandran plot, which implies the accuracy of model prediction (Supplementary Fig. 4B). Subsequently, the suitable Z-score value was obtained from *ProSA-web* evaluation (Supplementary Fig. 4C**)**, which is in the range of X-ray and NMR structure^58^ and QMEAN Z-Score from *SWISS Model Server* (Supplementary Fig. 4D**)** revealed that the 3D model is well fitted within the range of its authentic crystal structure conformation with the range validated structure^59^, indicating a very good agreement between the experimental and modelled structure. Furthermore, the overall energy of the model was discovered to be negative, which corresponds to proper and reliable regions (Supplementary Fig. 4E). Successively, the Levitt-Gerstein (LG) score and expected MaxSub of the Zfu2p model were investigated by the *ProQ tool*^60,61^. The LG score of > 5 (10.255) denotes that the predicted structure is of very good quality^60,61^. The similar conclusions were reached by utilizing the *ERRAT* plot, where the total quality factor is 91.3%, indicating that more than 91% of residues were detected under the error cutoff of 95%^62^ (Supplementary Fig. 4F).

### Virtual screening, drug likeness, inhibitory constant, and ligand-protein interactions analysis

**Fig. 5:**
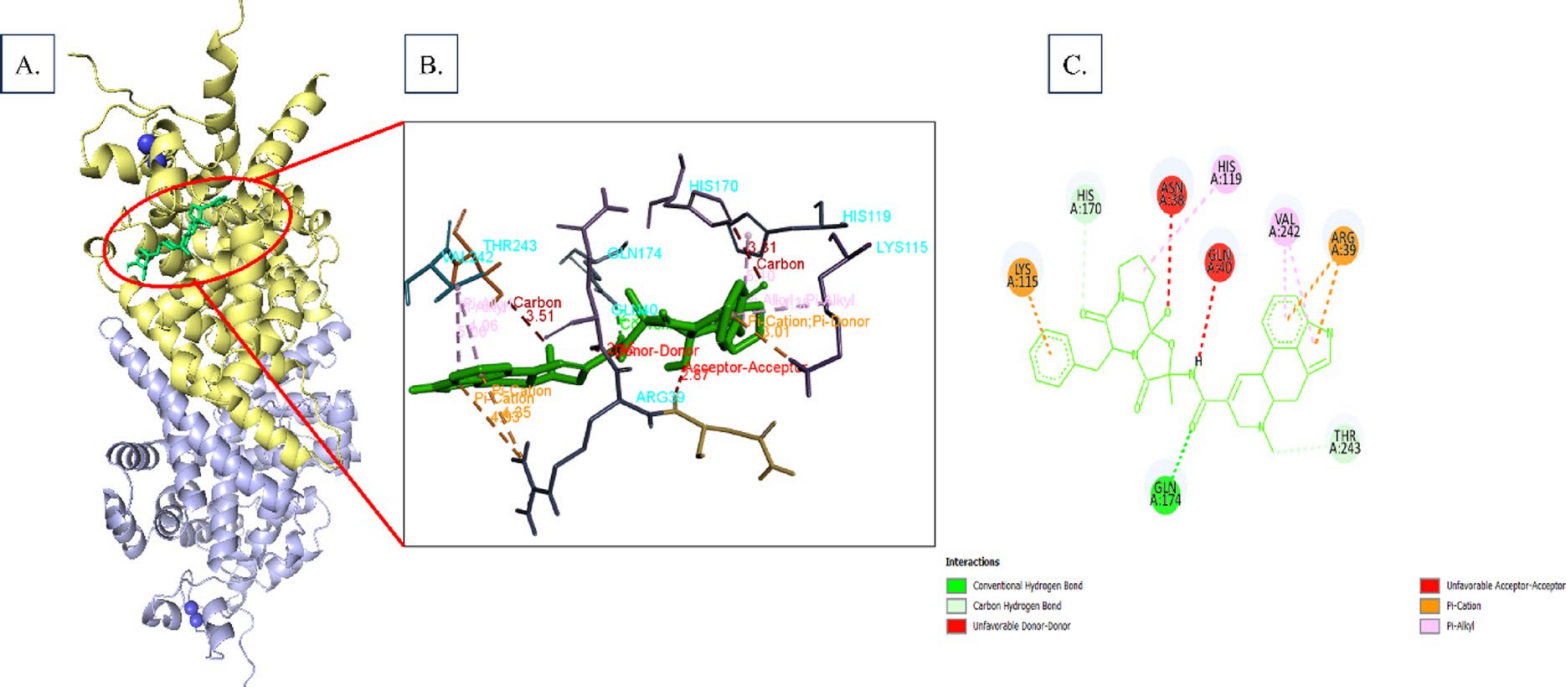
Molecular docking of (**A**) Ergotamine (Green), with homodimer subunit of Zfu2p (Yelllow and violet), with 4Zn^2+^ molecules (blue). (**B**) Magnified view of interaction site (**C**) 2D interaction visualization in ligand-binding site on Zfu2p.

Zfu2p contains two functional binding regions within amino acids 9–44, comprising a DNA-binding domain and a conserved zinc-binding site^52,53^. The conservation of this region suggests that it may serve as a potential ligand-binding site, making it a promising target for structure-based drug design and virtual screening studies. Afterward, we extracted 1614 FDA approved drugs from *ZINC15* database^63^ and went for LIPINSKI rule screening by applying ADMETlab 3.0^64^. After that we performed virtual screening against Zfu2p as a target and it was performed using AutoDock Vina in *PyRx 0.8*^65^ by toggling the amino acids (9–44) in prioritized stretch of target (Grid Box: centre_x= -12.48,centre_y= − 18.63, centre_z= − 14.13, size_x = 30.42, size_y = 34.46, size_z = 29.51), the grid box was set to cover the ligand-binding site which coincides the DNA and Zn^2+^ binding site. Based on Lipinski’s rule and inhibitory constant (Ki) ranking^66^, the top five repurposed drug candidates exhibited strong binding affinities in the nanomolar range. Ergotamine (ZINC000052955754) showed the highest affinity, with a binding energy of − 10.0 kcal/mol corresponding to a Ki of 4.67 × 10⁻⁸ M (46.7 nM). Irinotecan (ZINC000001612996) followed with a binding energy of − 9.9 kcal/mol and a Ki of 5.51 × 10⁻⁸ M (55.1 nM). Nilotinib (ZINC000006716957) demonstrated a binding energy of − 9.5 kcal/mol, yielding a Ki of 1.08 × 10⁻⁷ M (108 nM), while Fosaprepitant (ZINC000003939013) exhibited a binding energy of − 9.2 kcal/mol with a Ki of 2.1 × 10⁻⁷ M (213 nM). Eltrombopag (ZINC000011679756) also displayed favorable binding, with a binding energy of − 9.1 kcal/mol with 2.12 × 10⁻⁷ M (212 nM), supporting its potential inhibitory activity. Collectively, these findings highlight the identification of repurposable drug candidates with predicted nanomolar-scale inhibitory potency. Furthermore, we utilized Biovia Discovery Studio to visualize and determine the interacting residues profile of Zfu2p with highest binding affinity drug and deduced that 4 hydrogen, hydrophobic and 3 electrostatic bonds were enriched (Fig. 5 and Supplementary Table 3).

### Molecular dynamics study

**Fig. 6:**
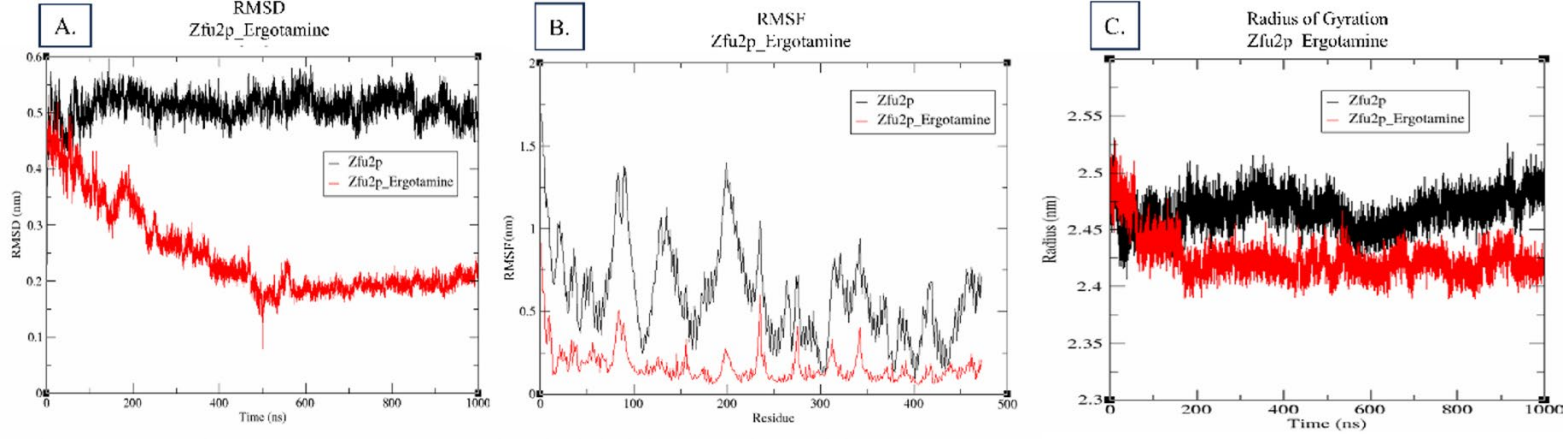
RMSD analysis of Zfu2p and Zfu2p-Ergotamine complex. (**A**) RMSD profile over 1000 ns illustrates structural stability of apo Zfu2p (black) and holo Zfu2p–Ergotamine complex (red). (**B**) RMSF analysis of Zfu2p and Zfu2p-Ergotamine complex. RMSF plots show residue-level flexibility of apo Zfu2p (black) compared with holo Zfu2p Ergotamine (red). (**C**) Rg analysis of Zfu2p and Zfu2p-Ergotamine complex. The Rg values reflect the overall compactness of the protein during the 1000 ns simulation.

To assess the stability and conformational behaviour of Zfu2p in the presence and absence of Ergotamine, molecular dynamics simulations were carried out for 1000 ns, and this time considered to be adequate and applied to study dimeric protein complex^67^. The Root Mean Square Deviation (RMSD) analysis revealed distinct stability patterns between the apo and holo systems. The apo Zfu2p exhibited higher structural deviations with an average RMSD of 0.51 nm, indicating larger conformational drift during the trajectory. In contrast, the Zfu2p-Ergotamine complex displayed a significantly reduced average RMSD of 0.24 nm, demonstrating that ligand binding stabilized the protein and restricted large-scale conformational rearrangements. This suggests that Ergotamine induces a rigid conformation through strong and stable interactions within the binding pocket (Fig. 6A).

The Root Mean Square Fluctuation (RMSF) profiles further supported this observation. Apo Zfu2p showed an average fluctuation of 0.60 nm, with pronounced peaks at loop regions, reflecting flexible residues stretch. Upon Ergotamine binding, the average RMSF decreased to 0.16 nm, particularly around the active-site residues, confirming reduced local flexibility and enhanced conformational stability of the holo complex (Fig. 6B).

The radius of gyration (Rg) was analyzed to evaluate the overall compactness of the protein structure during the simulation. The apo Zfu2p exhibited an average Rg of 2.46 nm, whereas the Zfu2p–Ergotamine complex showed a slightly lower Rg of 2.42 nm, suggesting a marginal increase in structural compactness upon ligand binding, while maintaining a stable structure after the 200 ns simulation.

This consistency indicates that Ergotamine binding preserved the protein’s tertiary structure without major unfolding events (Fig. 6C). Taken together, the MD simulation results demonstrate that Ergotamine significantly stabilizes Zfu2p by lowering both global (RMSD) and local (RMSF) fluctuations while maintaining overall compactness (Rg). This ligand-induced rigidification likely disrupts the native biological flexibility required for Zfu2p’s functional activity. Hence, Ergotamine binding may effectively inactivate Zfu2p, thereby impeding its contribution to VVC progression.

## Discussion

Vulvovaginal candidiasis is the paramount concern in the field of female health. The understanding till now is not adequate to tackle these opportunistic pathogens because of the perplexing ability to transform yeast form in commensalism to hyphal form in pathogenic conditions, and its evolution to impede drugs that we applied for treatment. The molecular pathophysiology of *C. albicans* and the host, during VVC and commensal lifestyle, is very intricate. We aim to dissect the molecules participating during both conditions to explore the scenario and to demarcate a regulatory molecule exclusively present in the pathogenic condition through a network biology approach, and generate a transcription factor based antifungal drug to circumvent the VVC.

We structured our study into four modules. First, we reconstructed condition-specific PPINs and GRNs using DEGs from disease (VVC) and healthy vaginal states. Second, we built host-pathogen interaction networks and mapped direct *C. albicans* interactors to generate pathogen-targeted networks, identifying key regulatory molecules. Third, functional enrichment analysis and literature validation were performed to elucidate the molecular mechanisms. Finally, we repurposed FDA-approved drugs against candidate pathogenic proteins and performed molecular dynamics analysis.

The reconstructed PPINs (Supplementary Fig. 1A and 1B) highlighted context specific molecular players associated with commensal and pathogenic states, enabling downstream GRN construction (Fig. 1A and 1B) respectively. Gene ontology analysis revealed that the pPPIN and pGRN was enriched in phenotypic switching and biofilm formation (Supplementary Fig. 2A and 2B), whereas the cPPIN and cGRN was enriched in iron homeostasis and symbiont-associated processes (Supplementary Fig. 2C and 2D), confirming network specificity.

We then generated two sub-networks: CTVPPIN (VVC state) and CTCPPIN (commensal state). CTVPPIN of host axis showed enrichment of pro-inflammatory pathways, including Th17 responses and adaptive immune signaling (Fig. 4A), while genes in pathogen axis show the enrichment of biofilm formation and its regulation, flocculation, regulation of phenotypic switching, host defence response, and ubiquitin-like protein binding function (Fig. 4B**).** In contrast, CTCPPIN highlighted innate immune functions maintaining host-microbe balance, including MAPK pathway components (Fig. 4C), while in the commensal axis of CTCPPIN, we observed that cytokinesis, regulation of growth, yeast-form cell wall, negative regulation of filamentous form, catabolite repression, and induction of host defence response by symbiont (Fig. 4D). It is worthwhile to mention that we did not receive significant pro-inflammatory response related functions in the pool of genes associated with commensalistic interactions, which provides us a pivotal clue to commensal environment establishment between host and opportunistic pathogen. Next, we analyzed pathogenic proteins from CTVPPIN within the pGRN and commensal proteins from CTCPPIN within the cGRN to trace their regulatory roles. Comparative analysis revealed Zfu2p as an exclusive master regulator in the pGRN, along with its associated transcription factors, all absent in the cGRN.

**Fig. 7:**
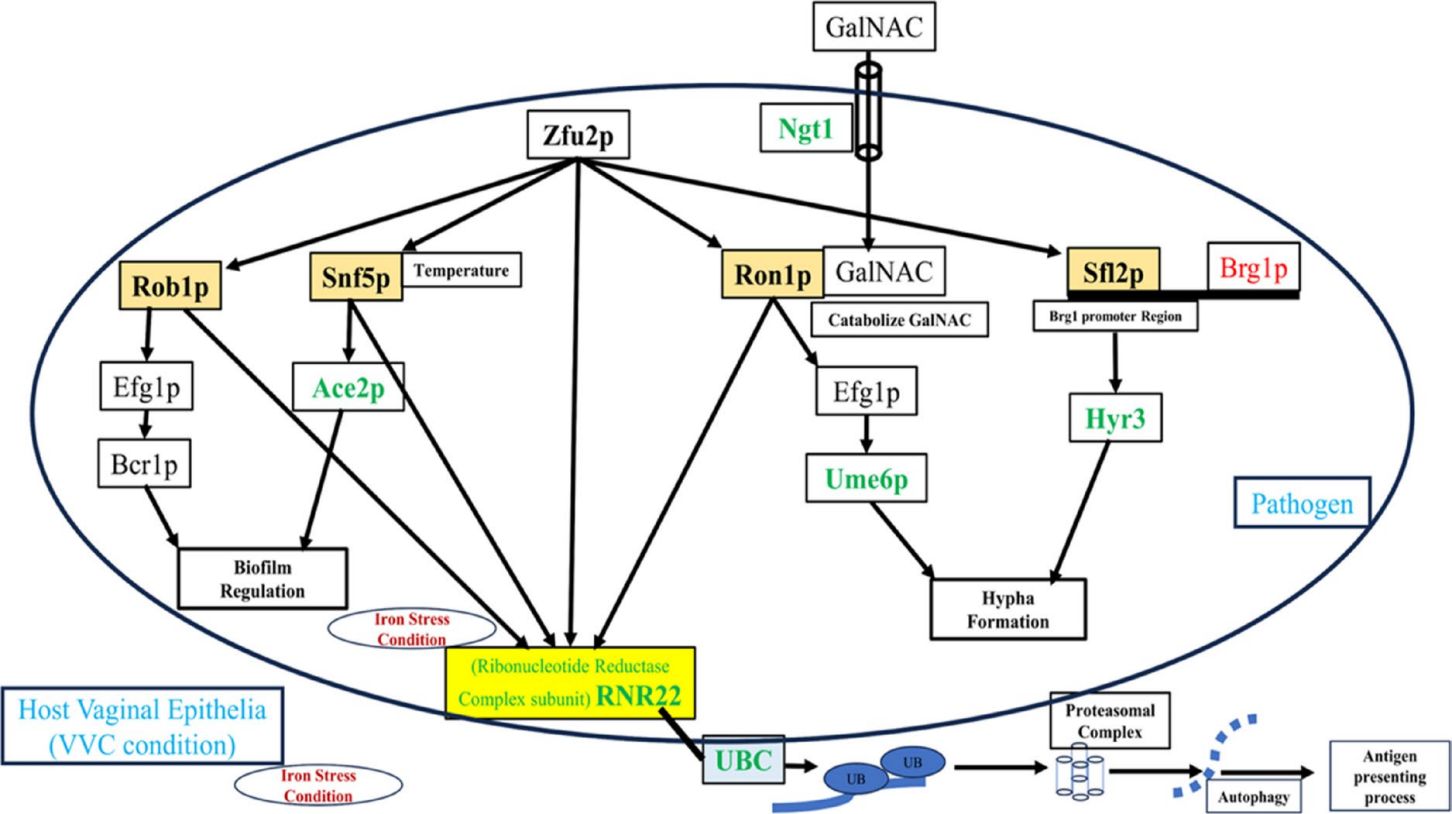
Diagrammatic representation of the Zfu2p pathogenic master regulator’s downstream cascade during VVC condition. Green color proteins represent upregulation, and red color protein represents downregulation during VVC. Black color bold protein represents pTFs.

Subsequently, from the interaction cascade of the Zfu2p in the pGRN network, we received some fascinating results. It was found to control 4 pTFs- Ron1p, Snf5p, Sfl2p, and Rob1p (Fig. 7). Literary evidence suggested that N-acetylglucosamine (GalNAC) is a triggering source for *C. albicans* to change its phenotypic structure from commensal to pathogenic form^68^. GalNAC is internalized by the transporter called Ngt1, which we observed to be upregulated in our analysis in pathogenic conditions (log2FC = 3.69). Afterwards, with the aid of Ron1p, GalNAC is catabolised and facilitates the interaction of Ron1p with Efg1p and Ume6p to form hyphae^68^. Eventually, Ume6p was also found to be upregulated in pathogenic conditions (log2FC = 2.47), which clearly suggests a crucial role of Ron1p in the establishment of their pathogenicity. Similarly, Snf5p is a chromatin remodeling factor that controls Ace2p (log2FC = 3.22), which in turn represses hyphae to yeast transformation^69^. Moreover, it was evidenced that Sfl2 gets activated by sensing the temperature and binds to the promoter region of Brg1p to repress it, which in turn activates the hypha specific gene Hyr3^70^. Accordingly, we also found the downregulation of Brg1p (log2FC=-2.08) and upregulation of Hyr3 (log2FC = 2.3) in the pathogenic condition. Additionally, it was proven that Rob1p interacts with Egf1p and Bcr1p to induce biofilm formation^71^. Therefore, our results clearly demonstrated that the four downstream TFs of Zfu2p, i.e., Ron1p, Snf5p, Sfl2p, and Rob1p, play imperative roles in hyphal formation through their interaction with other proteins **(**Fig. 7**)**. Thus, it is reasonable to target the master regulator Zfu2p for controlling the expression of these TFs.

Zfu2p was found to interact with the host polyubiquitin C (UBC) protein in CTVPPIN, facilitated by the stress-induced RNR22 (log2FC = 2.58) of the ribonucleotide reductase (Fig. 7) complex, which mitigates iron limitation imposed by the host (RNR1/RNR3 large subunits; RNR21/RNR22 small subunits)^68^. During pathogenic conditions, host iron-binding proteins such as lipocalin, lactoferrin, and calprotectin restrict pathogen growth^69^, generating oxidative stress^70^. RNR proteins counter this by arresting the cell cycle at G1/S^71^ and scavenging iron^68^. Iron stress also triggers ubiquitin-mediated proteolysis and autophagy^72^. In our analysis, RNR22 directly interacts with UBC, which connects with 422 of 466 proteins in CTVPPIN, and UBC itself is upregulated under VVC (log2FC = 1.34). This direct interaction of pathogenic protein RNR22 and host protein UBC might indicate a host mechanism to withhold pathogenesis during VVC.

Furthermore, mutational studies on Zfu2p conferred the evidence that mutation would make *C. albicans* unable to form biofilm^69,72^. Another mutational study of Zfu2p in the *G. mellonella* model had shown significantly low fungal burden^73^. Therefore, it could be stated that drug targeting against Zfu2p would be able to hamper the crucial pathogenic pathways, i.e., hyphal, biofilm formation, and iron scavenging process of *C. albicans* during VVC. Interestingly, earlier study by Boehm et al. demonstrated that Zfu2p acts as a repressor of filamentation in murine models, thereby promoting commensal colonization in specific host niches. However, commensal behavior observed in murine environments, such as the gastrointestinal tract, cannot be directly extrapolated to humans due to fundamental interspecies differences in immune architecture, epithelial signaling, microbiome composition, and metabolic landscapes that shape host-fungal interactions^74^. Notably, fungal pathogens elicit species-specific immune responses, with human immune cells exhibiting distinct patterns of antigen presentation, cytokine signaling, and pattern-recognition receptor regulation compared with murine systems^74^. Accordingly, whether Zfu2p plays a similar commensal regulatory role in the human gut remains unexplored. Hence, our findings suggest that targeting Zfu2p may attenuate fungal virulence without disrupting commensal behavior in the human vaginal niche.

Next, we have also performed multiple sequence alignment of Zfu2p (region: − 9–44) of *C. albicans* with other pathogens ortholog proteins, which is one of the primary causes of VVC and revealed that this region is also conserved in other pathogen as well (Supplementary Fig. 3), which strongly supports that targeting this region of Zfu2p protein could help to counter other pathogenic fungi which causes VVC.

Though it is a known fact that transcription factors have long been ignored as drug targets due to an inappropriately defined ligand binding site and considered as undruggable^75^, but TFs have a crucial function in binding to DNA and controlling the expression of target genes, playing vital roles in regulating various biological functions, which we also find in our study. Since numerous signalling pathways ultimately intersect at TFs, aiming treatments at these TFs might offer greater precision and fewer adverse effects compared to targeting the initial standard targets within these pathways. Therefore, considering all of these attributes collectively, TFs possess substantial promise and exceptional specificity as targets for therapeutic drugs. Nowadays, TFs are used and identified as a target for the treatment of cancer, protozoan parasites, and pharmacological manipulation^76–79^.

*C. albicans* is a member of the normal human microbiota, and it is also a causative agent of VVC in healthy women as well. The antifungal drugs that we were using to treat VVC or fungal infection are limited, toxic, and have a narrow spectrum of activity. One of the main reasons is prolonged use, which makes the pathogen adopt the resistant mechanism to that drug, and the second one is that it creates dysbiosis in microflora, which provokes imbalance in their commensal lifestyle, which aids VVC to a more severe condition. Candida infections are strongly linked to increasing rates of VVC and RVVC in pregnant and healthy women. Consequently, there is an urgent demand for new antifungal medications. Hence, we were able to establish an antifungal drug target through our meticulous protocol, which is exclusively present in pathogenic condition, i.e., Zfu2p. Therefore, in this study, we employed the drug repurposing method of FDA-approved drugs extracted from the ZINC15 database against Zfu2p for treatment during vulvovaginal candidiasis. We performed docking analysis, and LIPNSKI rule of five screening, which delivered the top five drugs. Ergotamine (ZINC000052955754) showed the highest affinity, with a binding energy of − 10.0 kcal/mol corresponding to a Ki of 4.67 × 10⁻⁸ M (46.7 nM). Thus, we prioritized Ergotamine as a highly effective drug repurposing against *C. albicans*. Subsequently, we proceed with MDS studies with the top-most drug, i.e., Ergotamine, targeting Zfu2p as a target in VVC. From our analysis, it was observed that the structural stability of the bound protein with the drug is rigid and stable compared to the apo form, which divulged the inhibition of Zfu2p functions.

Ergotamine has long been documented as an effective therapeutic agent for the treatment of migraine attacks^80^. However, despite its clinical efficacy, ergotamine is associated with significant adverse effects and contraindications. Its use is restricted in patients with peripheral vascular disease, severe or uncontrolled hypertension, ischemic heart disease, angina pectoris, sepsis, peptic ulcer disease, renal or hepatic impairment, porphyria, uterine contraction risk, overdose susceptibility, or known hypersensitivity to ergot alkaloids^81,82^. Management of ergotamine toxicity has been reported to involve vasodilatory agents such as sodium nitroprusside, which can mitigate vasospastic complications^83^. Importantly, concomitant administration of ergotamine with azole antifungals (e.g., itraconazole, posaconazole, and voriconazole) is contraindicated due to potent CYP3A4 inhibition. Since CYP3A4 is primarily responsible for ergotamine metabolism, its inhibition markedly increases systemic ergotamine levels, predisposing patients to ergotism^84^. Recent advances in analytical chemistry and biotechnology have enabled the characterization and development of ergot alkaloid analogs, including dihydro-ergot derivatives, which demonstrate reduced vasospastic and toxic effects compared to parent ergotamine^85,86^ In, thereby reducing the risk of pharmacokinetic interactions. Thus, with appropriate care and monitoring, ergotamine and its derivate can be an efficacious, safe, and cost-effective treatment.

Although we have successfully established the prominent molecular cross-talks during pathogenesis and commensal condition of *C. albicans* and identified a drug against the candidate protein. However, there are limitations to this approach, including dependence on homology and the inability to predict strain or lineage-specific protein interactions. Furthermore, the prevalent reliance on the SC5314 reference strain, which possesses unique characteristics limiting its representativeness^87–89^, further restricts broad biological insights. In contrast to laboratory wild-type strains, clinical isolates of *C. albicans* exhibit distinct phenotypic adaptations; including enhanced filamentation, stress tolerance, metabolic flexibility, and antifungal resistance-that reflect host-driven selective pressures encountered during infection. Thus, future studies incorporating transcriptional data from diverse clinical isolates derived from asymptomatic carriers and VVC patients will be essential to validate the generalizability of our observations. Additionally, as reported by Valentine et al. 2025 existing in vitro models cannot accurately recapitulate the transition from commensalism to pathogenicity or effectively model neutrophil dysfunction observed in vulvovaginal candidiasis^48,90^, resulting in a paucity of comprehensive omics data that capture these dynamic and context-dependent phenomena. Lactic acid tolerance differs among *C. albicans* strains, with SC5314 remaining resilient while other isolates are negatively impacted depending in the growth condition^91,92^. This underscores the need for more diverse isolates and complex experimental systems that combine vaginal epithelium and immune components to generate robust omics datasets for further analysis. Subsequently, the molecular target identified in this study was computationally predicted and would require additional experimental validation.

## Conclusion

In conclusion, our analysis of the integrated networks in VVC and the commensal state of both the host and pathogen underscores the critical role of Zfu2p in pathogenic attributes, which contributes to VVC. Targeting Zfu2p as an antifungal drug target presents a promising strategy for treating VVC, which may offer therapeutic benefits without disrupting the commensal balance of the vaginal microbiome. Moreover, after drug repurposing and MDS analysis, we proposed Ergotamine as a preferred treatment for VVC, supported by its LIPINSKI rule of 5, strong binding affinity, Ki value, and interaction analysis with drug. This approach holds potential for more effective management of VVC.

## Materials and methods

### Identification of differentially expressed genes of *C. albicans* and human during vulvovaginal candidiasis and commensal repertoire

Pathogenic genes linked to VVC were extracted from a supplementary of RNASeq data analysis by Manczinger et al., *GSE54694*^39^, where immortalized vaginal epithelial cells were interacted with live, yeast-like *C. albicans*. Meanwhile, it is a well-known fact that hyphal and biofilm forming genes of *C. albicans* are denoted as a symbol of establishing pathogenic repertoire^42,93,94^, and the prevalence of yeast form, adherence, and colonization in healthy asymptomatic humans represents a symbiotic or commensal lifestyle of *C. albicans* with the host^1^. So, after perusal of the condition, DEGs expressed during the development of *C. albicans* hyphae in the vicinity of vaginal epithelial cell line (PK E6/E7) from 0 to 3 hours’ time duration, were regarded as a state of pathogenicity. To investigate the commensal phase, we considered studying the commensalism stages, i.e., adhesion and colonization, which are paramount for *C. albicans* to sustain a commensal lifestyle in the vaginal repertoire. Since serum free complete keratinocyte medium (CKM) is a nutrient rich medium, designed to support the growth and maintenance of keratinocytes cells, including vaginal epithelial cells and utilized for analyzing adhesion and forces required for adhesion^95^, CKM is formulated to support both the growth of *C. albicans* and keratinocyte cells, enabling studies of fungal-host interactions under conditions that better replicate the natural environment on host epithelial surfaces. We retrieved commensal DEGs between the 0 h to 3 h time points of *C. albicans* grown in CKM. Since experimental datasets explicitly capturing the commensal phase are scarce, this comparison was used as a proxy mimicking the commensal gene expression state relevant to adhesion and colonization, but not to active infection or pathogenicity. This approach leverages the fact that CKM medium, optimized for keratinocyte culture, provides an environmental background simulating early fungal colonization dynamics essential for commensalism in the vaginal epithelium. We had screened *C. albicans* DEGs in both pathogenic and commensal repertoire by considering log2FC≥|1| and adj. P value < 0.05 **(**Supplementary Table 1**).**

For human DEGs, we stipulated a RNASeq dataset *GSE207081*^48^ in which monolayer of A-431 vaginal epithelial cells challenged with two distinct strains of *C. albicans*, VVC strain (CA01887) and colonizing strain (CA14314). After carefully deducing the experimental design and condition of the dataset, we determined that the pathogenic strain (CA01887) helps in establishing themselves in the vaginal region and we expected that proteins expressed in this event related to immune response by host to impede VVC infection while colonizing strain (CA14314) encouraged in establishing commensal repertoire in the vaginal region and we expected that proteins expressed in this event related to immune response by host was non-inflammatory, able to distinguish between the yeast and pathogenic form of *C. albicans* and able to tolerate the yeast cell growth. Using DESeq2 package in R programme v4.2.2, we analysed raw read counts. DESeq2 calculates DEGs by using negative binomial distribution^96^. The DEGs from RNASeq datasets for human were screened by applying log2FC≥|1| and adj. P value < 0.05 **(**Supplementary Table 2**).**

### Reconstruction of the pathogen and commensal condition integrated gene regulatory network of *C. albicans*

We employed *STRING v11.5*^40^ to build protein-protein interaction networks (PPIN) with DEGs of *C. albicans* in pathogenic and commensal conditions (pPPIN- pathogenic condition PPIN and cPPIN- commensal condition PPIN, respectively) (Supplementary Fig. 1A and Supplementary Fig. 1B). These networks contain only physical interactions with a cut-off of interaction confidence score ≥ 0.4^8^. We have utilized Cytoscape v3.10.0^97^ to visualize these networks. Furthermore, we also harnessed the *Pathoyeastract* database^98^ for predicting TF-TG interactions of 598 TGs of pPPIN in “biofilm-forming and numerous stress conditions”. For the commensal environment, we employ the “log phase growth” condition for predicting TF-TG interaction for 824 TGs. *Pathoyeastract* database contains experimentally validated (RNA-seq - WT vs. TF mutant, Microarray WT vs. TF mutant, chip on chip, ChIP), TF-TG association data^99^. For the extraction of pathogenic and commensal TF-TF interaction, we employed the *Pathoyeastract* database^98^ in biofilm-forming, other pathogen-promoting conditions, and log phase conditions, respectively. For TG-TG interaction for both conditions, we used *STRINGv11.5*^40^ with a cut-off of interaction confidence score ≥ 0.4.

### Calculation of the master regulator in transcriptional networks

We have constructed a hierarchical network of pGRN and cGRN by calculating the in-degree and out-degree of the intricate web of interactions (TF-TF, TF-TG, TG-TG) in the two transcriptional cascades by utilizing “Network Analyzer,” a Cytoscape v3.10.0 plugin^97^, and afterward we applied the previously devised protocol to obtain master regulators^100,101^. Then the hierarchy index (v), which ranges between − 1 and 1 for all the nodes, was calculated by the following formula.

1 $$\begin{aligned}\:v=\frac{(out\:degree-in\:degree)}{(out\:degree+in\:degree)}\end{aligned}$$

### Discerning host-pathogen interaction based on the interologs approach

We have used *HPIDB3.0*^102^ for predicting host-pathogen interolog. For the prediction of Human-*C. albicans* PPIs, we only considered those PPIs that were inferred from experiments such as Y2H, co-immunoprecipitation, and other experimentally robust protocols as templates. We have taken the interologs list of 119 host proteins and 317 *C. albicans* proteins, constituting 432 interactions, which was accomplished by screening with a minimum percentage identity of 50%, a minimum query coverage of 90%, and an E value threshold of ≤ 10^− 10^ for the blast homology search and GO function similarity from our previous research article^47^.

### Constructing vagina specific pathogen-targeted network of humans in both conditions

To construct vagina specific pathogen-targeted network, we followed a 3-step methodology. First, we took 6938 genes expressed in the vaginal tissue with a TPM value of ≥ 11 from Protein Atlas^103^ and reconstructed a vagina specific PPIN network (vPPIN) in *stringApp* in Cytoscape plugin v3.10.0^104^ by taking physical interaction and a cut-off score of interaction confidence score ≥ 0.4. Then, DEGs of both pathogenic and commensal conditions (established by VVC (CA01887) and colonizing (CA14314) strain, respectively), were extracted from the *GSE207081* dataset and mapped distinctly in vPPIN to procure the condition-specific sub-vPPIN network. Finally, we mapped the interolog interactions of the host protein pool that directly interacted with pathogen protein with condition-specific sub-vPPIN, for procuring condition-specific *C. albicans* target networks (CTVPPIN- *C. albicans* targeted VVC specific PPIN and CTCPPIN- *C. albicans* targeted commensal PPIN).

### Ontology analysis of inferred networks

We performed GO ontology enrichment analyses of pPPIN, cPPIN, pGRN, cGRN, CTVPPIN, and CTCPPIN of *C. albicans* using ClueGO database^41^, a Cytoscape plug-in v3.10.0^97^. Whereas, GO ontology enrichment analyses for host proteins (the target network of *C. albicans* in humans) were performed by using *Enrichr*^49^. ClueGO is a Cytoscape plugin, and Enrichr is a web-based enrichment analysis tool that offers an extensive selection of biological functional annotations to help researchers understand the biological significance of vast gene lists^41,49^. We considered only those GO biological, cellular, and molecular enrichment terms having an adj. P value < 0.05.

### Multiple sequence alignment and identification of potential ligand-binding sites

The Zfu2p protein of *C. albicans* and the protein sequence of 7 pathogenic species that are *Candida dubliniensis* CD36 (Cd36_87120), *Candida parapsilosis* CDC317 (CPAR2_602060), *Candida famata* (DEHA2D18810p), *Candida guilliermondii* (PGUG_05199), *Candida glabrata* CBS138 (CAGL0K11902g), *Candida auris* (B9J08_000794), and *Aspergillus nidulans* (AN5924) were procured from NCBI^105^ and are known to be the leading cause of VVC. The pairwise alignment and homology analysis were conducted by *Clustal Omega*^106^, and visualization was done using Esprit 3.0^107^. Furthermore, based on our network analysis, we determined that the Zfu2p protein was uniquely present in the pathogenic (VVC) condition, instigating a transcriptional cascade. Subsequently, we made a thorough analysis of the NCBI database and literature survey about Zfu2p, where we found that a specific region (9 to 44 amino acid residues) is responsible for its biological function^52,53^. Additionally, 6 cysteine residues in the above-mentioned region were conserved and not found in higher eukaryotes like humans as well. Furthermore, this specific region also contains two binding sites, i.e., DNA and Zn^2+^ binding sites^53^. These potential attributes strongly suggested that a stretch of 9–44 amino acid residues might be considered as a ligand binding site.

### Homology modelling and structure validation

Applying the Alpha Fold3 server, a three-dimensional homodimer structure with 4 Zn^2+^ ions was constructed of a prioritized protein sequence, i.e., Zfu2p. Afterward, it was employed in the GalaxyWeb^57^ for structural refinement. To substantiate the refined 3D structure of Zfu2p, we utilized a distinct set of online tools. Firstly, *PROCHECK*, which unravels the stereochemical characteristics (torsional angles-phi [ϕ] and psi [ψ]) with the help of the Ramachandran plot. We uploaded the Zfu2p refined structure file to the web servers to validate the model. The outputs include a number of charts in post script format as well as a thorough listing of each residue. The model was validated by analysing the Ramachandran plot in PROCHECK^108^. We again cross validated the result in the *SWISS-Model* server to validate the structure by Ramachandran plot^59^. Furthermore, we applied tools for quality and the least potential protein folding energy of the refined model through *ProSA* web (Protein Structure Analysis) and QMEAN in the *Swiss Model* server, respectively^59,109^. ProSA and QMEAN of the Swiss Model server calculate an overall quality score for a specific input structure and a composite scoring function assessing the major geometrical aspects of protein structures, respectively. After uploading the refined Zfu2p structure in pdb format, the Z-score value, and a plot of the residues’ energy as an output were procured from ProSA, and a QMEAN score and a plot were received from the Swiss Model Server after QMEAN analysis. Further, the *ERRAT* server, a program for confirming protein structures derived from crystallographic research and substantiating the structure based on patterns of nonbonded atomic interactions, was used to confirm the 3D conformation of the refined model. Error values are shown graphically as functions of a sliding nine-residue window’s position. The error function is calculated based on the statistics of nonbonded atom-atom interactions in the reported structure^62^. For a high-quality model, the accepted range is greater than 50%^110^. In addition, we utilized the *ProQ* tool, which employs a neural network incorporating various structural characteristics, to aid in evaluating the quality of a protein model based on either the LGscore or MaxSub criteria. Acceptable models should have an LGscore > 1.5 and a MaxSub value > 0.1^61^.

### Drug screening, docking and molecular dynamics simulation

The utilization of virtual screening techniques allows for the efficient filtering of molecules in the process of drug discovery. To accomplish this, we utilized PyRx 0.8, a software tool specifically designed for virtual screening tasks^65^. 1615 FDA approved drug compounds were obtained in 3D from the ZINC15 database and saved in a single file in SDF format. Subsequently, we also applied LIPINSKI rule of 5 for screening the drugs, by utilizing ADMETlab 3.0^64^. Subsequently, the SDF files were imported into PyRx’s Open Babel, and all the drugs underwent energy minimization using the “uff” force field (universal force field) and 200 minimization steps in PyRx, employing the conjugate gradient optimization algorithm. The drugs were then converted into AutoDock PDBQT format. Additionally, these compounds were subjected to docking using AutoDock Vina^111^ in PyRx 0.8, against the prioritized target protein that had been prepared. To encompass the ligand-binding site that corresponds to the functional domain of the target protein (specifically, the DNA and Zn^2+^ binding site of Zfu2p), by toggling 9 to 44 amino acid residues in the macromolecule structure and subsequently, we defined a grid box (Grid Box Dimension: - Centre_x= -12.42, Centre_y= -5.56, Centre_z= -43.51, Size_x = 36.51, Size_y = 34.53, Size_z = 30.33). We identified the top therapeutic compounds based on their therapeutic uses, considering their binding energies. Subsequently, we also determined inhibitory constant (K_i_), which is a specific type of equilibrium dissociation constant that quantifies the binding affinity between an inhibitor and its target enzyme or receptor^66^. It represents the concentration of inhibitor required to occupy half of the active sites of the enzyme in the absence of any competing ligands. A lower K_i_ value indicates a stronger binding affinity and therefore a more potent inhibitor. The docking scores obtained from the repurposed drugs with the target protein were converted into inhibitory constant (Ki) values using the established thermodynamic equation by Van ’t Hoff equation^112^.

2 $$\begin{aligned}\:Ki={e}^{\varDelta G/RT}\end{aligned}$$

ΔG is the binding free energy in kcal/mol (negative value), R is universal gas constant = 1.987*10^− 3^ kcal/molK, T is absolute temperature in Kelvin (usually 298.15 K), Ki is inhibitory constant in molar concentration (M).

For further analysis of the interaction profile, we utilized a cutting-edge tool called Biovia Discovery Studio. This tool designed to automatically detect and visualize relevant protein-ligand interactions in 3D structures. To gain insights, we focused on examining the interaction profiles of the top five hits using this tool.

Following molecular docking, molecular dynamics simulation is employed to assess the stability and behaviour of drugs in a dynamic biological environment. This step is evaluated for the flexibility and interactions of the drug within the biological system^113^, confirming its potential therapeutic efficacy for VVC and validating the stability of the drug protein complex. MDS was conducted using Gromacs version 2025.0 to assess the stability of the protein during the simulation. We performed MDS for both the apo and holo form of Zfu2p and the complex with Ergotamine. The Charmm36 force field was utilized, and the protein’s topology was generated via the CGenFF web server. Solvation was achieved with the protein centred in a dodecahedral box with a 1.0 nm minimum solute box distance and solvated with TIP3P water. The system was neutralized and brought to 0.1 M NaCl by replacing water molecules with Na⁺ and Cl⁻ ions, with the final ion counts determined by neutrality at the target concentration. The system was subjected to an energy minimization process with the steepest descent algorithm for 50,000 steps, followed by equilibration under NVT (number of molecules, volume, temperature) 1ns and NPT (number of molecules, pressure, temperature) 2ns and ensembles at 300 K and 1 bar pressure. Production MD was run for 1000 ns in the NPT ensemble at 300 K and 1 bar, using the leap-frog integrator with dt = 2 fs (nsteps = 50000000; 2 * 500000000 = 1000000 ps (1000ns or 1µs)). The simulation trajectory was calculated and analysed using RMSD, RMSF, Rg, and visualized in Graphing Advanced Computation and Exploration (XMGRACE).

**Fig. 8:**
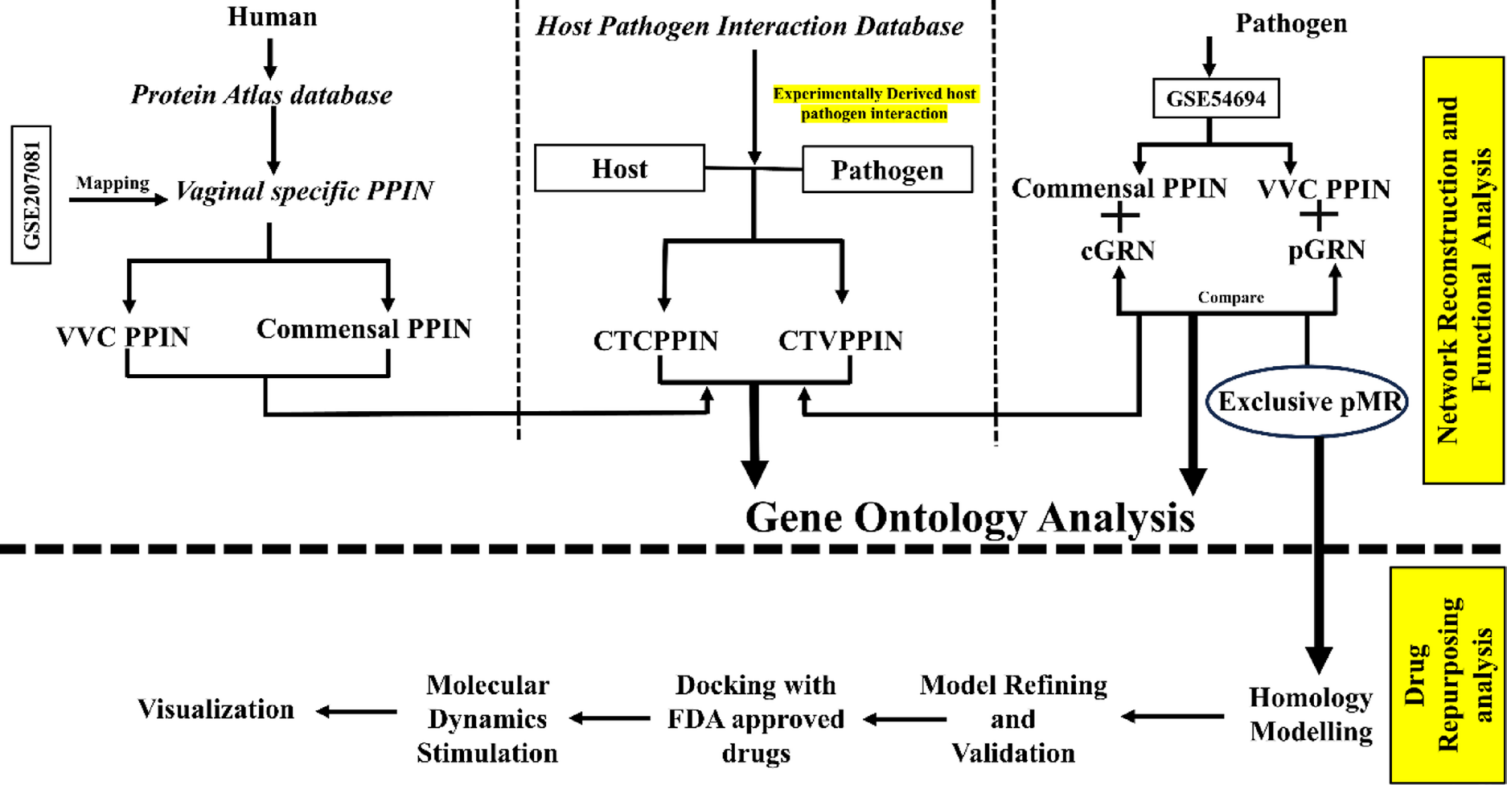
Methodology work flow.

The whole in-silico workflow has been portrayed in Fig. 8.

## Supplementary Information

Below is the link to the electronic supplementary material.

Supplementary Material 1

Supplementary Material 2

Supplementary Material 3

Supplementary Material 4

Supplementary Material 5

Supplementary Material 6

Supplementary Material 7

## Data Availability

Data utilized in this study, extracted from the NCBI GEO dataset, which is publicly available- GSE207081, GSE54694.

## Abbreviations

DEG: Differentially Expressed Genes
TF: Transcription Factor
PPIN: Protein-Protein Interaction Network
GRN: Gene Regulatory Network
RVVC: Recurrent Vulvovaginal Candidiasis
VVC: Vulvovaginal Candidiasis
vPPIN: vaginal specific PPIN
CTCPPIN: C. albicans targeted commensal PPIN
CTVPPIN: C. albicans targeted VVC specific PPIN

## References

[1] Jacobsen, I. D. The Role of Host and Fungal Factors in the Commensal-to-Pathogen Transition of Candida albicans. *Curr. Clin. Microbiol. Rep.* **10**, 55–65 (2023). 37151578 10.1007/s40588-023-00190-w PMC10154278

[2] Czechowicz, P., Nowicka, J. & Gościniak, G. Virulence Factors of Candida spp. and Host Immune Response Important in the Pathogenesis of Vulvovaginal Candidiasis. *Int. J. Mol. Sci.* **23** (11), 5895 (2022). 35682581 10.3390/ijms23115895 PMC9179972

[3] Mayer, F. L., Wilson, D. & Hube, B. Candida albicans pathogenicity mechanisms. *Virulence* **4**, 119–128 (2013). 23302789 10.4161/viru.22913 PMC3654610

[4] Chow, E. W. L., Pang, L. M. & Wang, Y. From Jekyll to Hyde: The Yeast–Hyphal Transition of Candida albicans. *Pathogens* 10, (2021). 10.3390/pathogens10070859 PMC8308684 34358008

[5] Denning, D. W., Kneale, M., Sobel, J. D. & Rautemaa-Richardson, R. Global burden of recurrent vulvovaginal candidiasis: a systematic review. *Lancet Infect. Dis.* **18**, e339–e347 (2018). 30078662 10.1016/S1473-3099(18)30103-8

[6] Ray, A., Aayilliath, K., Banerjee, A., Chakrabarti, S., Denning, D. W. & A. & Burden of Serious Fungal Infections in India. *Open. forum Infect. Dis.* **9**, ofac603 (2022). 36589484 10.1093/ofid/ofac603 PMC9792086

[7] Ardizzoni, A., Wheeler, R. T. & Pericolini, E. It Takes Two to Tango: How a Dysregulation of the Innate Immunity, Coupled With Candida Virulence, Triggers VVC Onset. *Front. Microbiol.* **12**, 692491 (2021). 34163460 10.3389/fmicb.2021.692491 PMC8215348

[8] Kabir, A. R. & Podder, S. An integrated bioinformatics and machine learning-based approach to depict key immunological players associated with candidemia during immunodeficiency. *Comput. Biol. Chem.* **119**, 108505 (2025). 40403354 10.1016/j.compbiolchem.2025.108505

[9] Bras, G. et al. Secreted Aspartic Proteinases: Key Factors in Candida Infections and Host-Pathogen Interactions. *Int. J. Mol. Sci.* **25** (9), 4775 (2024). 38731993 10.3390/ijms25094775 PMC11084781

[10] Roselletti, E. et al. NLRP3 inflammasome is a key player in human vulvovaginal disease caused by Candida albicans. *Sci. Rep.* **7**, 17877 (2017). 29259175 10.1038/s41598-017-17649-8 PMC5736597

[11] Cassone, A. Vulvovaginal Candida albicans infections: pathogenesis, immunity and vaccine prospects. *BJOG: Int. J. Obstet. \& Gynecol.* **122**, 785–794 (2015). 10.1111/1471-0528.12994 25052208

[12] Rodríguez-Cerdeira, C. et al. Pathogenesis and Clinical Relevance of Candida Biofilms in Vulvovaginal Candidiasis. *Front. Microbiol.* **11**, 544480 (2020). 33262741 10.3389/fmicb.2020.544480 PMC7686049

[13] He, Y., Liu, J., Chen, Y., Yan, L. & Wu, J. Neutrophil Extracellular Traps in Candida albicans Infection. *Front. Immunol.* **13**, 913028 (2022). 35784323 10.3389/fimmu.2022.913028 PMC9245010

[14] Apalata, T. & Longo-Mbenza, B. The Role of T Helper 17 (Th17) and Regulatory T Cells (Treg) in the Pathogenesis of Vulvovaginal Candidiasis among HIV-Infected Women. *International Journal of Microbiology* 8841113 (2020).

[15] Rosati, D., Bruno, M., Jaeger, M., ten Oever, J. & Netea, M. G. Recurrent Vulvovaginal Candidiasis: An Immunological Perspective. *Microorganisms* 8, (2020). 10.3390/microorganisms8020144 PMC7074770 31972980

[16] Yano, J., Peters, B. M., Noverr, M. C. & Fidel, P. L. Novel Mechanism behind the Immunopathogenesis of Vulvovaginal Candidiasis: Neutrophil Anergy. *Infect. Immun.* **86**, (2018). 10.1128/iai.00684-17 10.1128/IAI.00684-17 PMC5820946 29203543

[17] Miró, M. S. et al. Contribution of TLR2 pathway in the pathogenesis of vulvovaginal candidiasis. *Pathogens Disease*. **75**, ftx096 (2017). 10.1093/femspd/ftx096 28911197

[18] Shao, T. Y. et al. Commensal Candida albicans Positively Calibrates Systemic Th17 Immunological Responses. *Cell. Host Microbe*. **25**, 404–417e6 (2019). 30870622 10.1016/j.chom.2019.02.004 PMC6419754

[19] Markey, L. et al. Pre-colonization with the commensal fungus Candida albicans reduces murine susceptibility to Clostridium difficile infection. *Gut microbes*. **9**, 497–509 (2018). 29667487 10.1080/19490976.2018.1465158 PMC6287688

[20] Lopez-Medina, E. et al. Candida albicans Inhibits Pseudomonas aeruginosa Virulence through Suppression of Pyochelin and Pyoverdine Biosynthesis. *PLoS Pathog.* **11**, e1005129 (2015). 26313907 10.1371/journal.ppat.1005129 PMC4552174

[21] Netea, M. G., Quintin, J. & van der Meer, J. W. M. Trained immunity: a memory for innate host defense. *Cell. host microbe*. **9**, 355–361 (2011). 21575907 10.1016/j.chom.2011.04.006

[22] Cheng, S. C. et al. mTOR- and HIF-1α-mediated aerobic glycolysis as metabolic basis for trained immunity. *Sci. (New York N Y)*. **345**, 1250684 (2014). 10.1126/science.1250684 PMC4226238 25258083

[23] Tso, G. H. W. et al. Experimental evolution of a fungal pathogen into a gut symbiont. *Sci. (New York N Y)*. **362**, 589–595 (2018). 10.1126/science.aat0537 30385579

[24] Martín-Cruz, L. et al. Candida albicans V132 induces trained immunity and enhances the responses triggered by the polybacterial vaccine MV140 for genitourinary tract infections. *Front. Immunol.* **13**, 1066383 (2022). 36505433 10.3389/fimmu.2022.1066383 PMC9729253

[25] Quintin, J. et al. Candida albicans infection affords protection against reinfection via functional reprogramming of monocytes. *Cell. host microbe*. **12**, 223–232 (2012). 22901542 10.1016/j.chom.2012.06.006 PMC3864037

[26] Bistoni, F. et al. Evidence for macrophage-mediated protection against lethal Candida albicans infection. *Infect. Immun.* **51**, 668–674 (1986). 3943907 10.1128/iai.51.2.668-674.1986 PMC262402

[27] Krüger, W., Vielreicher, S., Kapitan, M., Jacobsen, I. D. & Niemiec, M. J. Fungal-Bacterial Interactions in Health and Disease. *Pathogens* 8, (2019). 10.3390/pathogens8020070 PMC6630686 31117285

[28] Spatz, M. et al. Antibiotic treatment using amoxicillin-clavulanic acid impairs gut mycobiota development through modification of the bacterial ecosystem. *Microbiome* **11**, 73 (2023). 37032359 10.1186/s40168-023-01516-y PMC10084673

[29] Singh, R., Bhardwaj, V. K., Das, P. & Purohit, R. A computational approach for rational discovery of inhibitors for non-structural protein 1 of SARS-CoV-2. *Comput. Biol. Med.* **135**, 104555 (2021). 34144270 10.1016/j.compbiomed.2021.104555 PMC8184359

[30] Bharadwaj, S. et al. Drug repurposing for ligand-induced rearrangement of Sirt2 active site-based inhibitors via molecular modeling and quantum mechanics calculations. *Sci. Rep.* **11**, 10169 (2021). 33986372 10.1038/s41598-021-89627-0 PMC8119977

[31] Bhardwaj, V. K. & Purohit, R. Targeting the protein-protein interface pocket of Aurora-A-TPX2 complex: rational drug design and validation. *J. Biomol. Struct. Dyn.* **39**, 3882–3891 (2021). 32448055 10.1080/07391102.2020.1772109

[32] Shirley, D. A., Sharma, I., Warren, C. A. & Moonah, S. Drug Repurposing of the Alcohol Abuse Medication Disulfiram as an Anti-Parasitic Agent. *Front. Cell. Infect. Microbiol.* **11**, 633194 (2021). 33777846 10.3389/fcimb.2021.633194 PMC7991622

[33] Miró-Canturri, A. et al. Repurposing of the Tamoxifen Metabolites to Combat Infections by Multidrug-Resistant Gram-Negative Bacilli. *Antibiot. (Basel Switzerland)*. **10** (3), 336 (2021). 10.3390/antibiotics10030336 PMC8004611 33810067

[34] Pires, D. et al. Repurposing Saquinavir for Host-Directed Therapy to Control Mycobacterium Tuberculosis Infection. *Front. Immunol.* **12**, 647728 (2021). 33841429 10.3389/fimmu.2021.647728 PMC8032898

[35] Rodrigo, C., Fernando, S. D. & Rajapakse, S. Clinical evidence for repurposing chloroquine and hydroxychloroquine as antiviral agents: a systematic review. *Clin. Microbiol. infection: official publication Eur. Soc. Clin. Microbiol. Infect. Dis.* **26**, 979–987 (2020). 10.1016/j.cmi.2020.05.016 PMC7250111 32470568

[36] Krajaejun, T. et al. The Repurposed Drug Disulfiram Inhibits Urease and Aldehyde Dehydrogenase and Prevents In Vitro Growth of the Oomycete Pythium insidiosum. *Antimicrob. agents Chemother.* **63** (8), e00609–e00619 (2019). 31138572 10.1128/AAC.00609-19 PMC6658754

[37] Sharma, A., Sharma, A., Rana, A. & Niraj, R. R. K. In-Silico Repurposing of Anticancer Drug (5-FU) as an Antimicrobial Agent Against Methicillin-Resistant Staphylococcus aureus (MRSA). *Int. J. Pept. Res. Ther.* **26**, 2137–2145 (2020).

[38] Rudrapal, M., Khairnar, S. J. & Jadhav, A. G. Drug Repurposing (DR): An Emerging Approach in Drug Discovery. in *Drug Repurposing* (ed. Badria, F. A.)IntechOpen, (2020). 10.5772/intechopen.93193

[39] Manczinger, M. et al. The Absence of N-Acetyl-D-glucosamine Causes Attenuation of Virulence of Candida albicans upon Interaction with Vaginal Epithelial Cells In Vitro. *Biomed. Res. Int.* **398045** (2015). 10.1155/2015/398045 PMC4558442 26366412

[40] Szklarczyk, D. et al. The STRING database in 2021: customizable protein–protein networks, and functional characterization of user-uploaded gene/measurement sets. *Nucleic Acids Res.* **49**, D605–D612 (2020). 10.1093/nar/gkaa1074 PMC7779004 33237311

[41] Bindea, G. et al. ClueGO: a Cytoscape plug-in to decipher functionally grouped gene ontology and pathway annotation networks. *Bioinf. (Oxford England)*. **25**, 1091–1093 (2009). 10.1093/bioinformatics/btp101 PMC2666812 19237447

[42] Harriott, M. M., Lilly, E. A., Rodriguez, T. E., Fidel, P. L. & Noverr, M. C. Candida albicans forms biofilms on the vaginal mucosa. *Microbiol. (Reading England)*. **156**, 3635–3644 (2010). 10.1099/mic.0.039354-0 PMC3068702 20705667

[43] Schrevens, S. et al. Methionine is required for cAMP-PKA mediated morphogenesis and virulence of Candida albicans. *Mol. Microbiol.* **109**, 415–416 (2018). 30187986 10.1111/mmi.14065

[44] Hall, R. A. et al. The Quorum-Sensing Molecules Farnesol/Homoserine Lactone and Dodecanol Operate via Distinct Modes of Action in Candida albicans. *Eukaryot. Cell*. **10**, 1034–1042 (2011). 21666074 10.1128/EC.05060-11 PMC3165441

[45] Jang, S. J., Lee, K., Kwon, B., You, H. J. & Ko, G. Vaginal lactobacilli inhibit growth and hyphae formation of Candida albicans. *Sci. Rep.* **9**, 8121 (2019). 31148560 10.1038/s41598-019-44579-4 PMC6544633

[46] Willems, H. M. E., Ahmed, S. S., Liu, J., Xu, Z. & Peters, B. M. Vulvovaginal Candidiasis: A Current Understanding and Burning Questions. *J. fungi (Basel Switzerland)*. **6** (1), 27 (2020). 10.3390/jof6010027 PMC7151053 32106438

[47] Kabir, A. R., Chaudhary, A. A., Aladwani, M. O. & Podder, S. Decoding the host–pathogen interspecies molecular crosstalk during oral candidiasis in humans: an in silico analysis. *Front. Genet.* **14**, 1245445 (2023). 37900175 10.3389/fgene.2023.1245445 PMC10603195

[48] Sala, A. et al. A New Phenotype in Candida-Epithelial Cell Interaction Distinguishes Colonization- versus Vulvovaginal Candidiasis-Associated Strains. *mBio* **14**, e0010723 (2023). 36856418 10.1128/mbio.00107-23 PMC10128025

[49] Xie, Z. et al. Gene Set Knowledge Discovery with Enrichr. *Curr. Protocols*. **1**, e90 (2021). 10.1002/cpz1.90 PMC8152575 33780170

[50] Richardson, J. P. & Moyes, D. L. Adaptive immune responses to Candida albicans infection. *Virulence* **6**, 327–337 (2015). 25607781 10.1080/21505594.2015.1004977 PMC4601188

[51] Moyes, D. L. et al. Candida albicans yeast and hyphae are discriminated by MAPK signaling in vaginal epithelial cells. *PloS one*. **6**, e26580 (2011). 22087232 10.1371/journal.pone.0026580 PMC3210759

[52] Todd, R. B. & Andrianopoulos, A. Evolution of a fungal regulatory gene family: the Zn(II)2Cys6 binuclear cluster DNA binding motif. *Fungal Genet. biology: FG B*. **21**, 388–405 (1997). 10.1006/fgbi.1997.0993 9290251

[53] MacPherson, S., Larochelle, M. & Turcotte, B. A fungal family of transcriptional regulators: the zinc cluster proteins. *Microbiol. Mol. biology reviews: MMBR*. **70**, 583–604 (2006). 10.1128/MMBR.00015-06 PMC1594591 16959962

[54] Tianqiao, S. et al. Genome-Wide Identification of Zn2Cys6 Class Fungal-Specific Transcription Factors (ZnFTFs) and Functional Analysis of UvZnFTF1 in Ustilaginoidea virens. *Rice Sci.* **28**, 567–578 (2021).

[55] Abramson, J. et al. Accurate structure prediction of biomolecular interactions with AlphaFold 3. *Nature* **630**, 493–500 (2024). 38718835 10.1038/s41586-024-07487-w PMC11168924

[56] David, A., Islam, S., Tankhilevich, E. & Sternberg, M. J. E. The AlphaFold Database of Protein Structures: A Biologist’s Guide. *J. Mol. Biol.* **434**, 167336 (2022). 34757056 10.1016/j.jmb.2021.167336 PMC8783046

[57] Ko, J., Park, H., Heo, L. & Seok, C. GalaxyWEB server for protein structure prediction and refinement. *Nucleic Acids Res.* **40**, W294–W297 (2012). 22649060 10.1093/nar/gks493 PMC3394311

[58] Agrawal, P., Thakur, Z. & Kulharia, M. Homology modeling and structural validation of tissue factor pathway inhibitor. *Bioinformation* **9**, 808–812 (2013). 24143050 10.6026/97320630009808 PMC3796881

[59] Waterhouse, A. et al. SWISS-MODEL: homology modelling of protein structures and complexes. *Nucleic Acids Res.* **46**, W296–W303 (2018). 29788355 10.1093/nar/gky427 PMC6030848

[60] Chakraborty, J., Maity, A. & Sarkar, H. A systematic drug repurposing approach to identify promising inhibitors from FDA-approved drugs against Nsp4 protein of SARS-CoV-2. *J. Biomol. Struct. Dyn.* **41**, 550–559 (2023). 34844509 10.1080/07391102.2021.2009033

[61] Wallner, B. & Elofsson, A. Can correct protein models be identified? *Protein science: publication Protein Soc.* **12**, 1073–1086 (2003). 10.1110/ps.0236803 PMC2323877 12717029

[62] Colovos, C. & Yeates, T. O. Verification of protein structures: Patterns of nonbonded atomic interactions. *Protein Sci.* **2**, 1511–1519 (1993). 8401235 10.1002/pro.5560020916 PMC2142462

[63] Sterling, T. & Irwin, J. J. ZINC 15 – Ligand Discovery for Everyone. *J. Chem. Inf. Model.* **55**, 2324–2337 (2015). 26479676 10.1021/acs.jcim.5b00559 PMC4658288

[64] Fu, L. et al. ADMETlab 3.0: an updated comprehensive online ADMET prediction platform enhanced with broader coverage, improved performance, API functionality and decision support. *Nucleic Acids Res.* **52**, W422–W431 (2024). 38572755 10.1093/nar/gkae236 PMC11223840

[65] Dallakyan, S. & Olson, A. J. Small-molecule library screening by docking with PyRx. *Methods Mol. biology (Clifton N J)*. **1263**, 243–250 (2015). 10.1007/978-1-4939-2269-7_19 25618350

[66] Yung-Chi, C. & Prusoff, W. H. Relationship between the inhibition constant (KI) and the concentration of inhibitor which causes 50 per cent inhibition (I50) of an enzymatic reaction. *Biochem. Pharmacol.* **22**, 3099–3108 (1973). 4202581 10.1016/0006-2952(73)90196-2

[67] Shen, L. et al. Probing the Druggablility on the Interface of the Protein–Protein Interaction and Its Allosteric Regulation Mechanism on the Drug Screening for the CXCR4 Homodimer. *Front. Pharmacol.* **10**, 1310 (2019). 31787895 10.3389/fphar.2019.01310 PMC6855241

[68] Naseem, S., Min, K., Spitzer, D., Gardin, J. & Konopka, J. B. Regulation of Hyphal Growth and N-Acetylglucosamine Catabolism by Two Transcription Factors in Candida albicans. *Genetics* **206**, 299–314 (2017). 28348062 10.1534/genetics.117.201491 PMC5419476

[69] Finkel, J. S. et al. Portrait of Candida albicans adherence regulators. *PLoS Pathog.* **8**, e1002525 (2012). 22359502 10.1371/journal.ppat.1002525 PMC3280983

[70] Znaidi, S., Nesseir, A., Chauvel, M. & Rossignol, T. d’Enfert, C. A comprehensive functional portrait of two heat shock factor-type transcriptional regulators involved in Candida albicans morphogenesis and virulence. *PLoS Pathog.* **9**, e1003519 (2013). 23966855 10.1371/journal.ppat.1003519 PMC3744398

[71] Fox, E. P. et al. An expanded regulatory network temporally controls Candida albicans biofilm formation. *Mol. Microbiol.* **96**, 1226–1239 (2015). 25784162 10.1111/mmi.13002 PMC4464956

[72] Chong, P. P. et al. Transcriptomic and Genomic Approaches for Unravelling Candida albicans Biofilm Formation and Drug Resistance—An Update. *Genes* **9**, (2018). 10.3390/genes9110540 PMC6266447 30405082

[73] Amorim-Vaz, S., Delarze, E., Ischer, F., Sanglard, D. & Coste, A. T. Examining the virulence of Candida albicans transcription factor mutants using Galleria mellonella and mouse infection models. *Front. Microbiol.* **6**, 367 (2015). 25999923 10.3389/fmicb.2015.00367 PMC4419840

[74] Hellmann, A. M. et al. Human and Murine Innate Immune Cell Populations Display Common and Distinct Response Patterns during Their In Vitro Interaction with the Pathogenic Mold Aspergillus fumigatus. *Front. Immunol.* **8**, 1716 (2017). 29270175 10.3389/fimmu.2017.01716 PMC5723658

[75] Zhuang, J., Liu, Q., Wu, D. & Tie, L. Current strategies and progress for targeting the undruggable transcription factors. *Acta Pharmacol. Sin.* **43**, 2474–2481 (2022). 35132191 10.1038/s41401-021-00852-9 PMC9525275

[76] Walters, H. A. & Temesvari, L. A. Target acquired: transcriptional regulators as drug targets for protozoan parasites. *Int. J. Parasitol.* **51**, 599–611 (2021). 33722681 10.1016/j.ijpara.2020.12.007 PMC8169582

[77] Fontaine, F., Overman, J. & François, M. Pharmacological manipulation of transcription factor protein-protein interactions: opportunities and obstacles. *Cell. Regeneration*. **4**, 2 (2015). 25848531 10.1186/s13619-015-0015-x PMC4365538

[78] Lambert, M., Jambon, S. & Depauw, S. & David-Cordonnier, M.-H. Targeting Transcription Factors for Cancer Treatment. *Molecules* 23, (2018). 10.3390/molecules23061479 PMC6100431 29921764

[79] Xiong, M., Li, B., Zhu, Q., Wang, Y. X. & Zhang, H. Y. Identification of transcription factors for drug-associated gene modules and biomedical implications. *Bioinformatics* **30**, 305–309 (2014). 24262216 10.1093/bioinformatics/btt683

[80] Tfelt-Hansen, P. et al. Ergotamine in the acute treatment of migraine: A review and European consensus. *Brain* **123**, 9–18 (2000). 10611116 10.1093/brain/123.1.9

[81] Ngo, M. & Tadi, P. Ergotamine/Caffeine. in (2023).

[82] Mulac, D. & Humpf, H. U. Cytotoxicity and accumulation of ergot alkaloids in human primary cells. *Toxicology* **282**, 112–121 (2011). 21295106 10.1016/j.tox.2011.01.019

[83] Skowronski, G. A., Tronson, M. D. & Parkin, W. G. Successful treatment of ergotamine poisoning with sodium nitroprusside. *Med. J. Australia*. **2**, 8–9 (1979). 502953 10.5694/j.1326-5377.1979.tb112646.x

[84] Eljaaly, K., Alshehri, S., Bhattacharjee, S., Al-Tawfiq, J. A. & Patanwala, A. E. Contraindicated drug–drug interactions associated with oral antimicrobial agents prescribed in the ambulatory care setting in the United States. *Clin. Microbiol. Infect.* **25**, 620–622 (2019). 30107284 10.1016/j.cmi.2018.08.002

[85] Schiff, P. L. Ergot and its alkaloids. *Am. J. Pharm. Educ.* **70**, 98 (2006). 17149427 10.5688/aj700598 PMC1637017

[86] Klotz, J. L. Global Impact of Ergot Alkaloids. *Toxins* **14** (3), 186 (2022). 35324683 10.3390/toxins14030186 PMC8949401

[87] Glazier, V. E. et al. The Candida albicans reference strain SC5314 contains a rare, dominant allele of the transcription factor Rob1 that modulates filamentation, biofilm formation, and oral commensalism. *mBio* **14**, e0152123 (2023). 37737633 10.1128/mbio.01521-23 PMC10653842

[88] Lohse, M. B., Ziv, N. & Johnson, A. D. Variation in transcription regulator expression underlies differences in white-opaque switching between the SC5314 reference strain and the majority of Candida albicans clinical isolates. *Genetics* **225** (3), iyad162 (2023). 37811798 10.1093/genetics/iyad162 PMC10627253

[89] Iracane, E. et al. Identification of an active RNAi pathway in Candida albicans. *Proc. Natl. Acad. Sci. U.S.A.* **121**, e2315926121 (2024). 38625945 10.1073/pnas.2315926121 PMC11047096

[90] Valentine, M., Wilson, D., Gresnigt, M. S. & Hube, B. Vaginal Candida albicans infections: host–pathogen–microbiome interactions. *FEMS Microbiol. Rev.* **49**, fuaf013 (2025). 40347186 10.1093/femsre/fuaf013 PMC12071381

[91] Lourenço, A., Pedro, N. A., Salazar, S. B. & Mira, N. P. Effect of Acetic Acid and Lactic Acid at Low pH in Growth and Azole Resistance of Candida albicans and Candida glabrata. *Front. Microbiol.* **9**, 3265 (2018). 30671051 10.3389/fmicb.2018.03265 PMC6331520

[92] Zangl, I. et al. Human Pathogenic Candida Species Respond Distinctively to Lactic Acid Stress. *J. fungi (Basel Switzerland)*. **6** (4), 348 (2020). 10.3390/jof6040348 PMC7762603 33302409

[93] Rodríguez-Cerdeira, C. et al. Biofilms and vulvovaginal candidiasis. *Colloids Surf., B*. **174**, 110–125 (2019). 10.1016/j.colsurfb.2018.11.011 30447520

[94] Wu, X. et al. Biofilm Formation of Candida albicans Facilitates Fungal Infiltration and Persister Cell Formation in Vaginal Candidiasis. *Front. Microbiol.* **11**, 1117 (2020). 32582081 10.3389/fmicb.2020.01117 PMC7289921

[95] Younes, J. A. et al. Vaginal epithelial cells regulate membrane adhesiveness to co-ordinate bacterial adhesion. *Cell. Microbiol.* **18**, 605–614 (2016). 26477544 10.1111/cmi.12537

[96] Sarkar, S. & Podder, S. Profiling the Braak progression in Parkinson’s disease: a transcriptomics and ML driven identification of progressive biomarker and prognostic ceRNA signature. *Neuroscience* **593**, 41–55 (2026). 41360364 10.1016/j.neuroscience.2025.12.011

[97] Assenov, Y., Ramírez, F., Schelhorn, S. E., Lengauer, T. & Albrecht, M. Computing topological parameters of biological networks. *Bioinf. (Oxford England)*. **24**, 282–284 (2008). 10.1093/bioinformatics/btm554 18006545

[98] Teixeira, M. C. et al. YEASTRACT+: a portal for the exploitation of global transcription regulation and metabolic model data in yeast biotechnology and pathogenesis. *Nucleic Acids Res.* **51**, D785–D791 (2022). 10.1093/nar/gkac1041 PMC9825512 36350610

[99] Monteiro, P. T. et al. YEASTRACT+: a portal for cross-species comparative genomics of transcription regulation in yeasts. *Nucleic Acids Res.* **48**, D642–D649 (2020). 31586406 10.1093/nar/gkz859 PMC6943032

[100] KABIR, A. L. I., Das, D. & Podder, S. Impairment of host adaptive immune response to Candida albicans infection: an in-silico insight on SARS-CoV-2 conspiracy. (2022). 10.21203/rs.3.rs-1644512/v1

[101] Yu, H. & Gerstein, M. Genomic analysis of the hierarchical structure of regulatory networks. *Proc. Natl. Acad. Sci. U.S.A.* **103**, 14724–14731 (2006). 17003135 10.1073/pnas.0508637103 PMC1595419

[102] Ammari, M. G., Gresham, C. R., McCarthy, F. M. & Nanduri, B. HPIDB 2.0: a curated database for host–pathogen interactions. *Database* (2016). 10.1093/database/baw103 PMC4930832 27374121

[103] Thul, P. J. & Lindskog, C. The human protein atlas: A spatial map of the human proteome. *Protein science: publication Protein Soc.* **27**, 233–244 (2018). 10.1002/pro.3307 PMC5734309 28940711

[104] Shannon, P. et al. Cytoscape: a software environment for integrated models of biomolecular interaction networks. *Genome Res.* **13**, 2498–2504 (2003). 14597658 10.1101/gr.1239303 PMC403769

[105] Sayers, E. W. et al. Database resources of the National Center for Biotechnology Information. *Nucleic Acids Res.* **47**, D23–D28 (2018). 10.1093/nar/gky1069 PMC6323993 30395293

[106] Sievers, F. & Higgins, D. G. Clustal Omega for making accurate alignments of many protein sequences. *Protein science: publication Protein Soc.* **27**, 135–145 (2018). 10.1002/pro.3290 PMC5734385 28884485

[107] Robert, X. & Gouet, P. Deciphering key features in protein structures with the new ENDscript server. *Nucleic Acids Res.* **42**, W320–W324 (2014). 24753421 10.1093/nar/gku316 PMC4086106

[108] Laskowski, R. A., MacArthur, M. W., Moss, D. S. & Thornton, J. M. {\it PROCHECK}: a program to check the stereochemical quality of protein structures. *J. Appl. Crystallogr.* **26**, 283–291 (1993).

[109] Wiederstein, M. & Sippl, M. J. ProSA-web: interactive web service for the recognition of errors in three-dimensional structures of proteins. *Nucleic Acids Res.* **35**, W407–W410 (2007). 17517781 10.1093/nar/gkm290 PMC1933241

[110] Messaoudi, A., Belguith, H. & Ben Hamida, J. Homology modeling and virtual screening approaches to identify potent inhibitors of VEB-1 β-lactamase. *Theor. Biol. Med. Model.* **10**, 22 (2013). 23547944 10.1186/1742-4682-10-22 PMC3668210

[111] Trott, O., Olson, A. J., AutoDock & Vina Improving the speed and accuracy of docking with a new scoring function, efficient optimization, and multithreading. *J. Comput. Chem.* **31**, 455–461 (2010). 19499576 10.1002/jcc.21334 PMC3041641

[112] Goettig, P., Chen, X. & Harris, J. M. Correlation of Experimental and Calculated Inhibition Constants of Protease Inhibitor Complexes. *Int. J. Mol. Sci.* **25** (4), 2429 (2024). 38397107 10.3390/ijms25042429 PMC10889394

[113] Karplus, M. & Petsko, G. A. Molecular dynamics simulations in biology. *Nature* **347**, 631–639 (1990). 2215695 10.1038/347631a0

