# Supplementary figures and images for: Devising a Zfu2p targeted antifungal strategy that may preserve commensalism while suppressing Candida albicans virulence during vulvovaginal candidiasis
Source: Sci Rep. 2026 May 13;16:19341. doi: 10.1038/s41598-026-46848-5 (PMC13287575; doi:10.1038/s41598-026-46848-5)

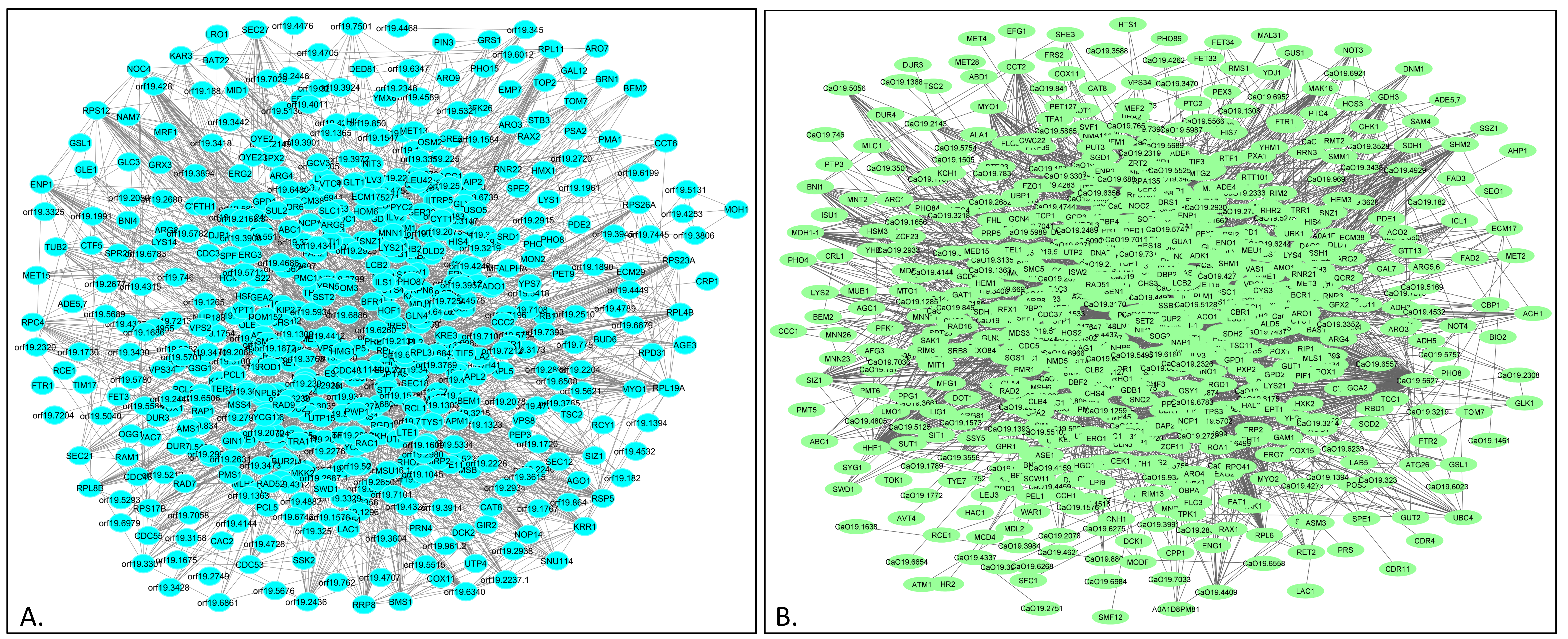

Supplement: Supplementary file 4 — Supplementary Material 4 [file 41598_2026_46848_MOESM4_ESM.tif]

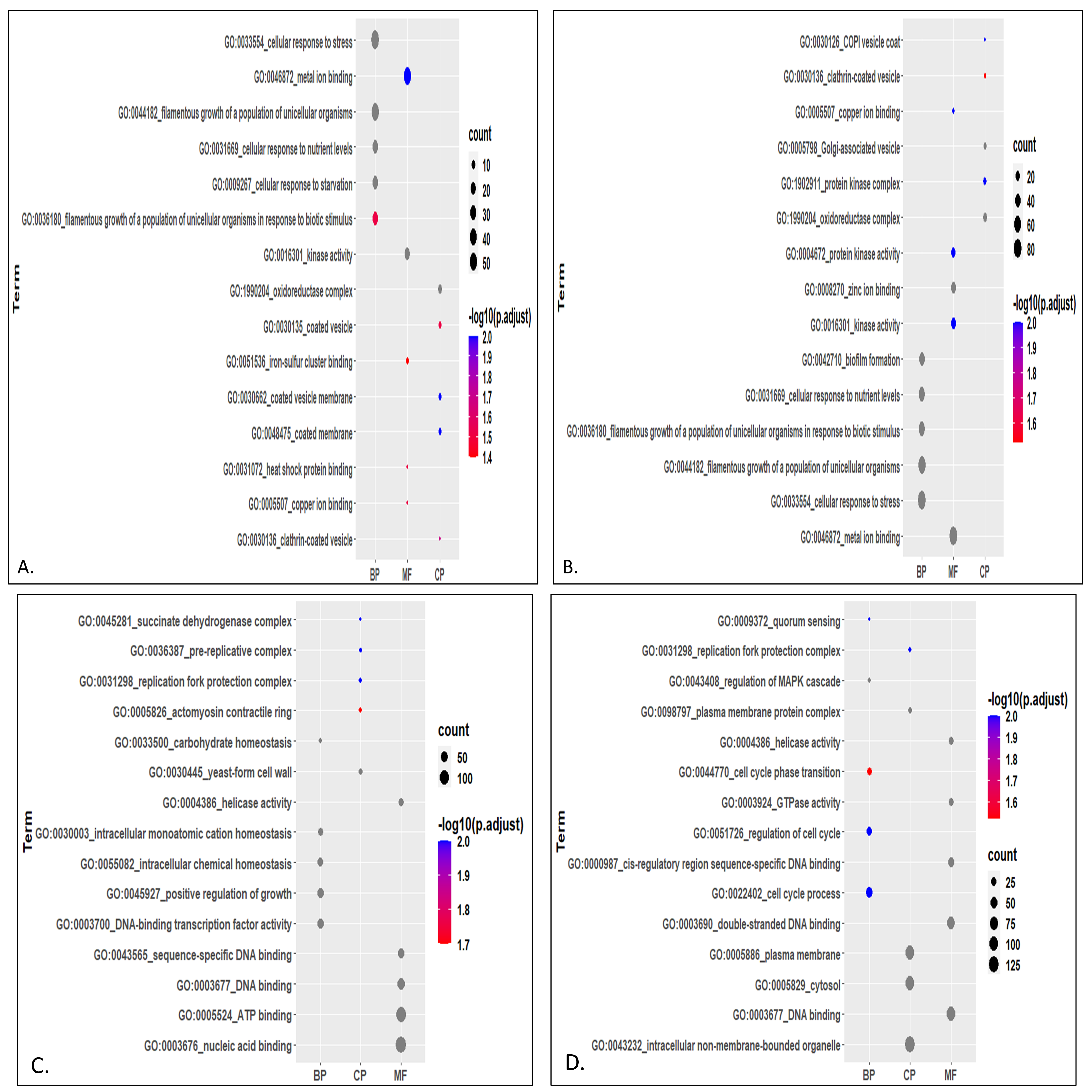

Supplement: Supplementary file 5 — Supplementary Material 5 [file 41598_2026_46848_MOESM5_ESM.tif]

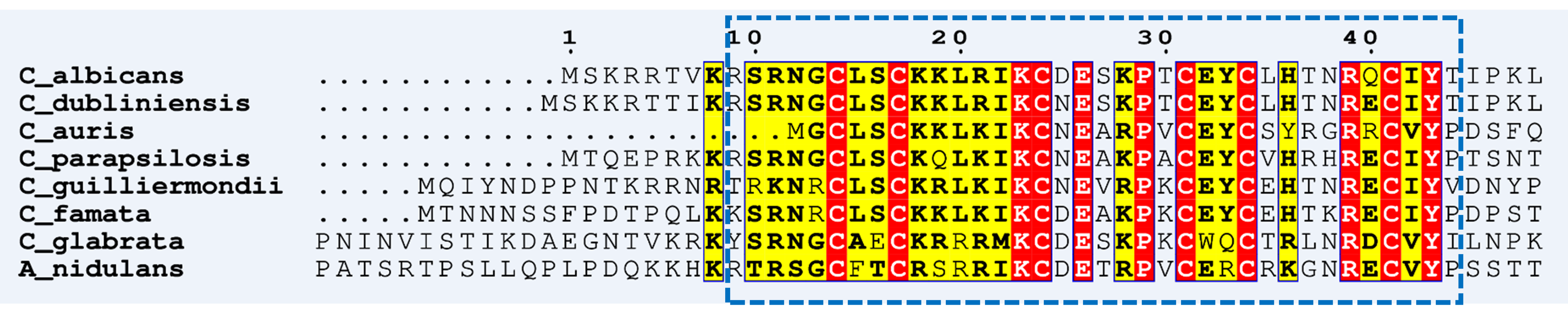

Supplement: Supplementary file 6 — Supplementary Material 6 [file 41598_2026_46848_MOESM6_ESM.tif]
